# Behavioral Abnormalities, Cognitive Impairments, Synaptic Deficits, and Gene Replacement Therapy in a CRISPR Engineered Rat Model of 5p15.2 Deletion Associated With Cri du Chat Syndrome

**DOI:** 10.1002/advs.202415224

**Published:** 2025-02-18

**Authors:** Jingjing Shen, Yan Wang, Yang Liu, Junying Lan, Shuang Long, Yingbo Li, Di Chen, Peng Yu, Jing Zhao, Yongjun Wang, Shali Wang, Feng Yang

**Affiliations:** ^1^ Institute of Neuroscience School of Basic Medicine Chongqing Medical University Chongqing 400016 China; ^2^ China National Clinical Research Center for Neurological Diseases Beijing Tiantan Hospital Capital Medical University Beijing 100070 China; ^3^ Basic and Translational Medicine Center China National Clinical Research Center for Neurological Diseases Beijing Tiantan Hospital Capital Medical University Beijing 100070 China; ^4^ Advanced Innovation Center for Human Brain Protection Capital Medical University Beijing 100070 China; ^5^ Laboratory of Cognitive and Behavioral Disorders Beijing Institute of Brain Disorders Capital Medical University Beijing 100069 China; ^6^ Chinese Institutes for Medical Research Capital Medical University Beijing 100069 China; ^7^ Department of Neurology Beijing Tiantan Hospital Capital Medical University Beijing 100070 China; ^8^ Clinical Center for Precision Medicine in Stroke Capital Medical University Beijing 100070 China; ^9^ Center of Excellence for Omics Research (CORe) Beijing Tiantan Hospital Capital Medical University Beijing 100070 China

**Keywords:** 5p15.2 deletion, Cri du Chat syndrome, *Ctnnd2*, gene therapy, rat model

## Abstract

The Cri du Chat Syndrome (CdCS), a devastating genetic disorder caused by a deletion on chromosome 5p, faces challenges in finding effective treatments and accurate animal models. Using CRISPR‐Cas9, a novel CdCS rat model with a 2q22 deletion is developed, mirroring a common genetic alteration in CdCS patients. This model exhibits pronounced deficits in social behavior, cognition, and anxiety, accompanied by neuronal abnormalities and immune dysregulation in key brain regions such as the hippocampus and medial prefrontal cortex (mPFC). The immunostaining and RNA‐seq analyses provide new insights into CdCS pathogenesis, revealing inflammatory and immune processes. Importantly, it is demonstrated that early gene replacement therapy with AAV‐*Ctnnd2* alleviates cognitive impairments in CdCS rats, highlighting the potential for early intervention. However, the effectiveness of this therapy is confined to the early developmental stages and does not fully restore all CdCS symptoms. The findings deepen the understanding of CdCS pathogenesis and suggest promising therapeutic directions.

## Graphical abstract

A new CdCS rat model is developed using CRISPR‐Cas9 technology. It shows impairments in cognitive and social behaviors, neuronal dendrite abnormalities in the hippocampus and mPFC, and inflammatory and immune processes in mPFC brain cells. Early AAV‐*Ctnnd2* gene therapy enhances cognitive function in CdCS rats.

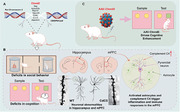

## 1 Introduction

Cri du Chat Syndrome (CdCS), also known as 5p minus syndrome, is a prevalent chromosomal deletion disorder caused by a heterozygous deletion on chromosome 5p.^[^ ^1^ ^]^ It affects 1 in 15 000 to 50 000 live births^[^ ^2^ ^]^ and presents with distinct symptoms such as a high‐pitched cry, microcephaly, facial abnormalities, and severe psychomotor and cognitive retardation.^[^ ^1^, ^3^ ^]^ The deletions can range from 5 to 40 Mb, with the 5p15.2 locus being closely associated with CdCS's hallmark features.^[^ ^4^ ^]^ While targeted therapies are limited, early rehabilitative and educational interventions show promise in improving prognosis and social integration for affected individuals.^[^ ^1^ ^]^

δ‐catenin, encoded by the *CTNND2* gene at 5p15.2, is the largest gene on chromosome 5p and crucial for cerebral development. Deletion of this gene in CdCS is linked to mental retardation. Studies show that *CTNND2* deficiency causes developmental delay, intellectual disability, and a significant link to autism spectrum disorder (ASD).^[^ ^5^ ^]^ The ASD‐associated G34S mutation disrupts social function by altering glutamatergic activity, but GSK3β inhibition can reverse synaptic and behavioral deficits.^[^ ^5^ ^f]^ Our research found that mice with a *Ctnnd2* mutation exhibit sleep disruptions and autism‐like behaviors,^[^ ^6^ ^]^ highlighting *CTNND2*’s key role in developmental disorders and autism risk.

Despite progress in understanding specific pathogenic mechanisms, pursuing disease‐modifying treatments for CdCS has yielded limited success. This challenge can be attributed to various factors, with a significant hurdle being the scarcity of animal models faithfully replicating the disease's pathogenesis for evaluating experimental treatments. Over decades, several *Ctnnd2* knockout mouse models have been developed.^[^ ^3^, ^5^, ^6^, ^7^ ^]^ However, none of these models have authentically replicated the key neuropathological traits observed in individuals with CdCS. This disparity may contribute to the scarcity of effective therapies for CdCS. In comparison to mice, genetic manipulation techniques in rats have advanced somewhat more slowly due to a lack of necessary tools.^[^ ^8^ ^]^ Nevertheless, rats exhibit greater physiological and behavioral resemblance to humans. Their larger size facilitates surgical manipulations and sample collection, such as blood/CSF samples.^[^ ^8a^ ^]^ Importantly, the genomic similarity between rats and humans suggests that modeling CdCS in rats could offer a more relevant avenue.

Globally, over 300 million individuals grapple with rare diseases, and a staggering 90% of these lack approved therapies. About 80% of these rare diseases stem from gene mutations.^[^ ^9^ ^]^ For decades, researchers have advocated for genetic modifications as promising remedies for various hereditary disorders, offering lasting and potentially curative clinical benefits through a single intervention.^[^ ^10^ ^]^ Using the adeno‐associated virus (AAV) for gene therapy presents an optimal approach for addressing hereditary ailments due to the non‐integrative nature of the genetic material delivered by this vector and exhibits low immunogenicity.^[^ ^10^ ^]^ Presently, five treatments have gained approval: Luxturna, Zolgensma, CAR‐T therapies (Yescarta and Kymriah), and Strimvelis.^[^ ^10^ ^]^ Other treatments for Hemophilia B and β‐Thalassemia are undergoing clinical trials.^[^ ^11^ ^]^ Recent discoveries highlight the efficiency of AAV‐PHP.eB in transducing brains, suggesting its potential utility in addressing neurological disorders.^[^ ^10^, ^12^ ^]^

Thus, an urgent necessity emerges to create a rat model that faithfully reproduces the predominant pathogenic mechanisms driving CdCS, utilizing the precision of CRISPR‐Cas9 gene editing technology. This study presents a successful rat model that meticulously mirrors a prevalent genetic anomaly found in CdCS. This achievement is attained through precise genetic deletion within the corresponding region, resulting in a rat model encompassing a comprehensive array of CdCS‐associated traits. These traits parallel those observed in genuine CdCS patients.

Concurrently, the pursuit of a gene replacement therapy tailored for the CdCS rat model holds immense promise. A groundbreaking gene replacement strategy, employing an adeno‐associated virus vector, is elucidated. This vector has been intricately engineered with unmatched precision to ensure an extensive and pervasive expression of *Ctnnd2* across the entire brain of the CdCS rat model. This strategic approach is firmly rooted in the reality that *CTNND2* holds the highest coverage frequency among genes within the deleted segment of the 5p region. This attribute holds true not just for individuals affected by Cri du Chat syndrome but also for the CdCS rat model, where the deletion of the *Ctnnd2* gene within the heterozygous 2q22 deletion accurately parallels the human condition.

**Figure 1:**
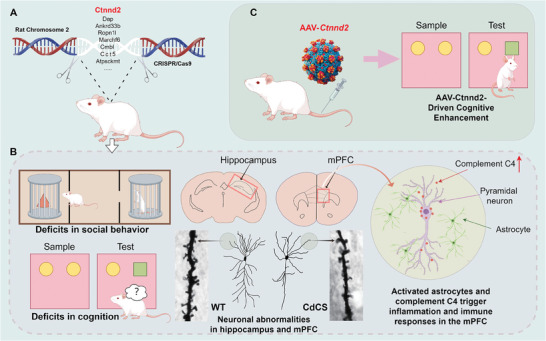
Schematic representation of a novel CdCS rat model created using CRISPR‐Cas9 technique, highlighting abnormal phenotypes and targeted gene therapy. A) A new CdCS rat model is developed utilizing the CRISPR‐Cas9 technique. B) This model demonstrates impairments in cognitive and social behaviors (left panel), along with significant abnormalities in neuronal dendrites within key brain regions, such as the hippocampus and mPFC (middle panel). In addition, inflammatory and immune processes within the brain cells of the mPFC are also evident (right panel). C) Early administration of AAV‐*Ctnnd2* gene replacement therapy enhances cognitive function in CdCS rats.

Engineered to enable widespread *Ctnnd2* expression within the brain of the CdCS rat model, this vector adeptly overcomes the blood–brain barrier (BBB). The result is a robust correction of deficiencies in both social behavior and cognitive function. These compelling and unequivocal outcomes powerfully underscore the substantial potential embedded in gene replacement therapy, offering a promising and feasible approach to tackle the intricate challenges presented by Cri du Chat Syndrome (**Figure** **1**).

## 2 Results

### 2.1 Generation of 5p15.2 Deletion on SD Rats

**Figure 2:**
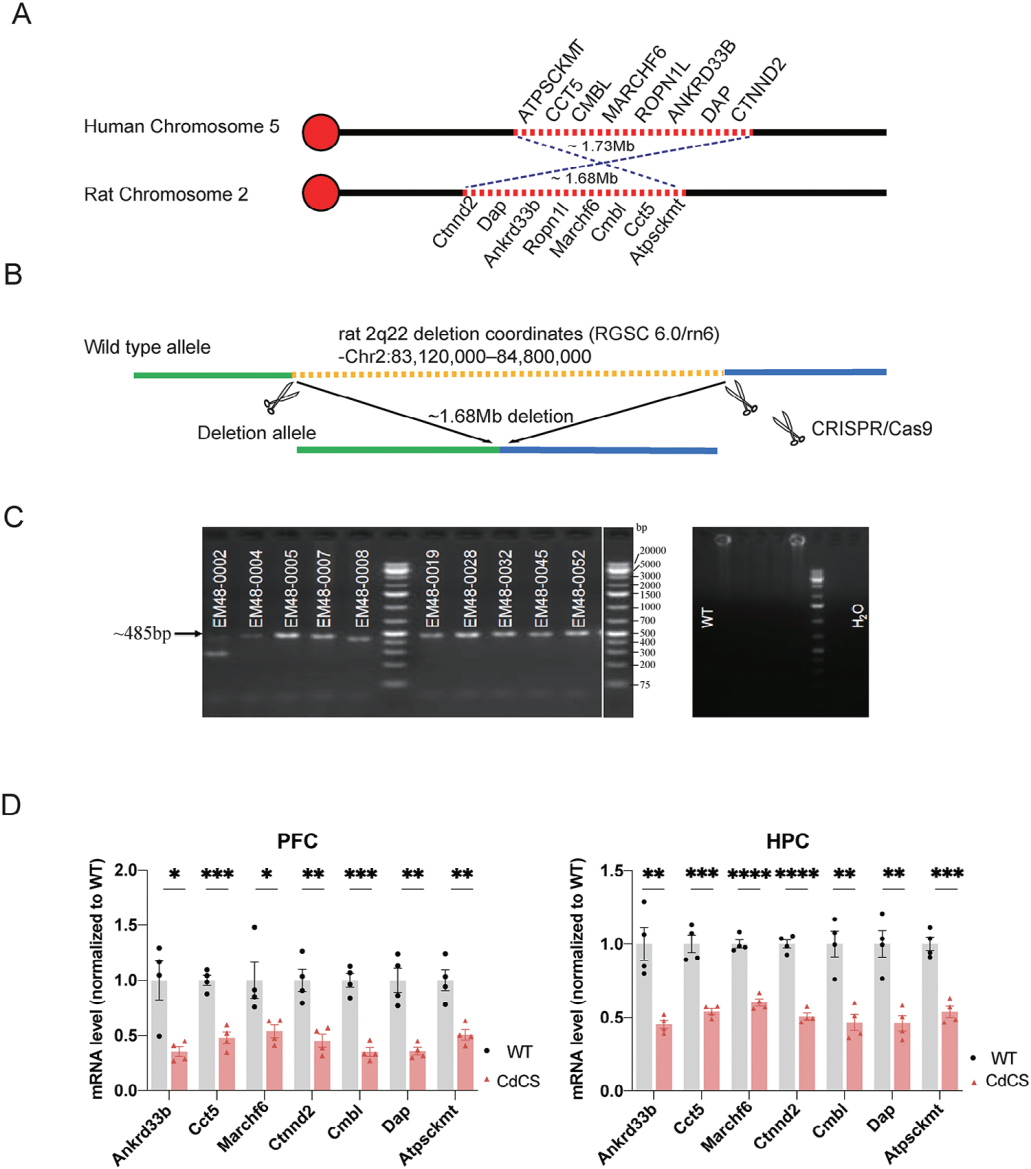
Generation of 5p15.2 deletion on the SD (Sprague Dawley) background using CRISPR/Cas9. A) The 5p15.2 region was located on human chromosome 5 (recurrent deletion coordinates (GRCh38/hg38)‐Chr5:10200000–11933334), and the syntenic 5p15.2 region was located on rat chromosome 2 (rat 2q22 deletion coordinates (RGSC 6.0/rn6)‐Chr2:83120000–84800000). B) Two Cas9/sgRNAs were designed (CRISPR‐A [Chr.2:83120000] and CRISPR‐B [Chr.2:84800000]) to create a ≈1.68Mb chromosomal deletion, a heterozygous 2q22 deletion. The deletion was confirmed using PCR. C) Confirmation of positive genotyping in the ten founder pups through PCR product sequencing, including #EM48‐0002, #EM48‐0004, #EM48‐0005, #EM48‐0007, #EM48‐0008, #EM48‐0019, #EM48‐0028, #EM48‐0032, #EM48‐0045, and #EM48‐0052. Due to space constraints, it was challenging to label the sixth lane with “bp”. Therefore, the molecular weight labeling for the sixth lane had been placed in the final lane (12th lane, left panel) for clarity. Please note that the content of the last lane was identical to that of the sixth lane and had been included solely to facilitate the labeling of the DNA molecular weight. D) The heterozygous rats with deletion of 2q22 had ≈50% decreased gene expression in most of the genes within the syntenic 5p15.2 region, including *Ctnnd2*, *Dap*, *Ankrd33b*, *Marchf6*, *Cmb1*, *Cct5*, and *Atpsckmt*. All genes were significantly reduced in both PFC and HPC (*p < 0.05, **p < 0.01, ***p < 0.001, and ****p < 0.0001) (*N*  =  four/group 2F + 2 M; three technical replicates per sample). Results represent mean ± SEM.

To generate rats that recapitulated the 5p15.2 deletion, we leveraged several features of the syntenic region on rat chromosome 2. The rat and human regions are nearly identical, with all eight genes arranged in the same order, except that the rat syntenic region is inverted compared to the human region. In addition, the rat region is slightly smaller, measuring 1.68 Mb compared to the human region's 1.73 Mb (**Figure** **2**A). To create a rat model of Cri du Chat syndrome, we mimicked the human 5p15.2 deletion by designing two sgRNAs (CRISPR‐A [Chr.2:83120000] and CRISPR‐B [Chr.2:84800000]) to induce an ≈1.68 Mb chromosomal deletion, a heterozygous 2q22 deletion (Figure 2B; Figure S1, Supporting Information). We performed PCR to confirm the presence of the heterozygous deletion in ten potential founder animals (Figure 2C). Subsequently, we conducted reverse transcriptase‐quantitative PCR (RT‐qPCR) to investigate whether the deletion of the syntenic region on rat chromosome 2q22 resulted in a diminished gene expression level in both the prefrontal cortex (PFC) and the hippocampus (HPC). The results confirmed that heterozygous rats with the 2q22 deletion exhibited ≈50% decreased gene expression in most of the genes within the syntenic 5p15.2 region, including *Ctnnd2*, *Dap*, *Ankrd33b*, *Marchf6*, *Cmb1*, *Cct5*, and *Atpsckmt* (Figure 2D). Notably, *Ropn1l* was not detected as its expression was specifically confined to the testis. These data demonstrate that the 2q22 deletion rat model faithfully recapitulated the genetic lesion observed in 5p15.2 deletion Cri du Chat syndrome. Therefore, we defined 2q22 deletion rat model as Cri du Chat syndrome rat model (also called CdCS rat model). Moreover, our CdCS rat model offered significant insights into potential phenotypic driver genes, providing a valuable resource for further investigation.

### 2.2 Growth Deficits and Delayed Nervous System Development

**Figure 3:**
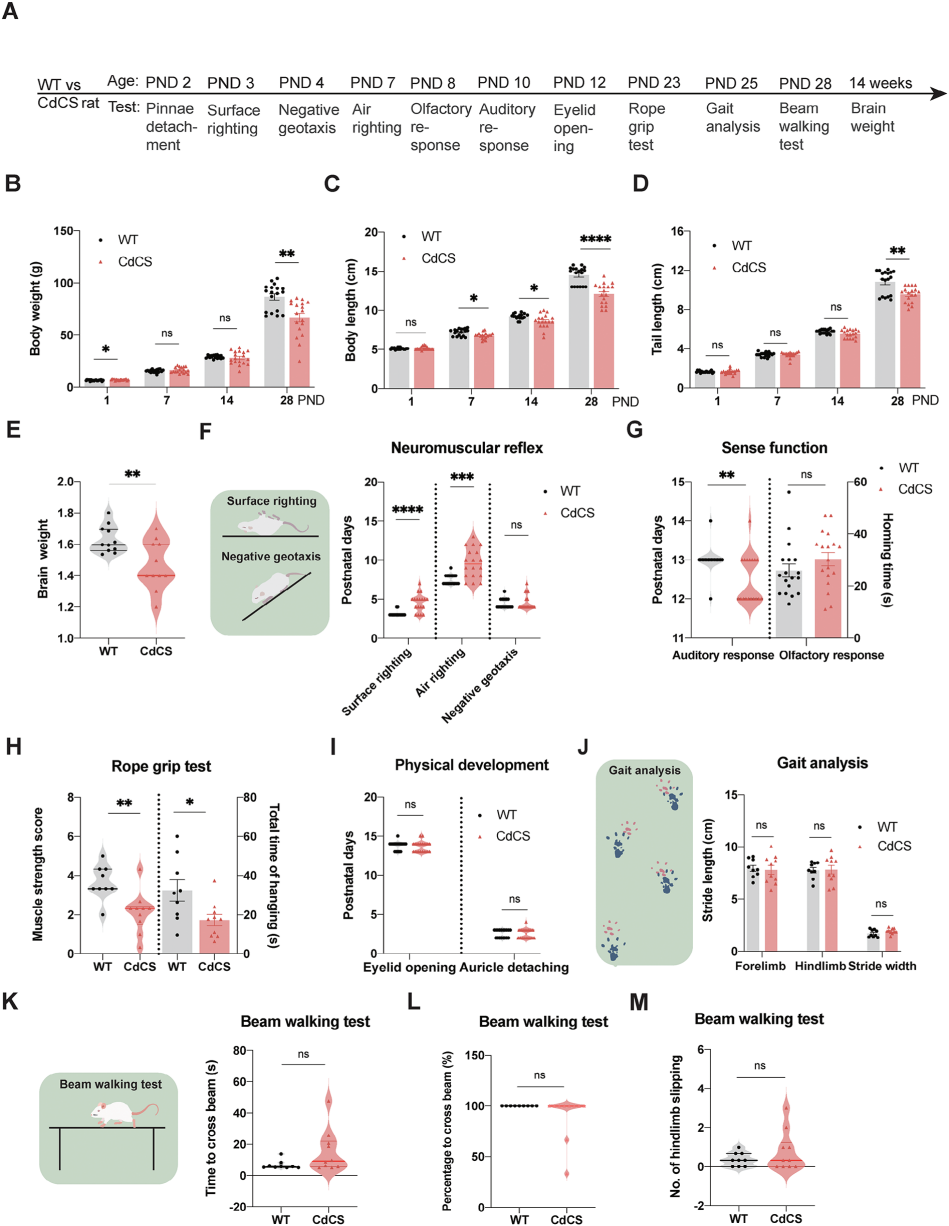
Growth deficits and delayed nervous system development in the CdCS rats. A) Experimental timeline and procedures for WT and CdCS rats. B–D) CdCS rats exhibited marked retardation in growth and development (*N* = 18/group, 9F + 9 M). Specifically: reduced body weight (B) and shorter body and tail lengths (C). D) Compared to WT rats, with statistical significance indicated (*p < 0.05 to ****p < 0.0001, ns = non‐significant). E) Brain weight significantly decreased in CdCS rats (*N* = 11/group, 4F + 7 M WT, 5F + 6 M CdCS). Mean values: WT = 1.63 ± 0.03 g, CdCS = 1.47 ± 0.05 g; **p < 0.01 (unpaired *t*‐test). F) Schematic diagram of neuromuscular reflex test (left panel). Delayed neuromuscular reflex development in CdCS rats (*N* = 18/group, 9F + 9 M). Significant delays in surface and air righting reflexes, but no difference in negative geotaxis (right panel). Statistical significance: ***p < 0.001 and ****p < 0.0001, ns = non‐significant (Mann–Whitney test, two‐tailed). Mean values: surface righting (WT: 3.11 ± 0.08 days, CdCS: 4.56 ± 0.27 days); air righting (WT: 7.50 ± 0.15 days, CdCS: 9.61 ± 0.45 days). Negative geotaxis (WT: 4.44 ± 0.15 days, CdCS: 4.61 ± 0.23 days). G) CdCS rats exhibited precocious auditory response (WT: 13.00 ± 0.08 days vs CdCS: 12.50 ± 0.15 days, **p < 0.01, Mann–Whitney) but no difference in olfactory response (*p* > 0.05). *N* = 18/group, 9F + 9M. H) CdCS rats had reduced muscle strength score (WT: 3.66 ± 0.29 vs CdCS: 2.13 ± 0.33, *p < 0.05, *t*‐test) and hanging time (WT: 32.37 ± 5.43s vs CdCS: 17.23 ± 2.9s, **p < 0.01, *t*‐test). [*N* = 9 WT (5F + 4 M), 10 CdCS (5F + 5 M)]. I) Normal eyelid opening and pinnae detachment in both groups (*N* = 18/group, 9F + 9 M). No statistical significance (Mann–Whitney test, *p* > 0.05). J) Schematic diagram of gait analysis (left panel). No difference in gait analysis between groups [*N* = 9 WT (5F + 4 M), 10 CdCS (5F + 5 M)]. No statistical significance (*t*‐test, *p* > 0.05) for forelimb, hindlimb stride length, and stride width (right panel). K–M) Schematic diagram of beam walking test (Figure 6K, left panel). CdCS rats showed normal balance capacity in beam walking test [*N* = 9 WT(5F + 4 M), 10 CdCS (5F + 5 M)]. No statistical significance for time to cross beam (Mann–Whitney test, *p* = 0.0966), passing percentage (Mann‐Whitney test, *p* = 0.4737), or the numbers of hindlimb slipping (Mann–Whitney test, *p* = 0.7166).

Subjects carrying the 5p15.2 deletion exhibited diminished birth weight, and this trend might endure during childhood.^[^ ^1^, ^13^ ^]^ To ascertain whether CdCS rats manifest a growth phenotype, we established cohorts of both female and male SD rats alongside wild‐type (WT) littermate controls (**Figure** **3**A). We conducted weekly assessments of rat body weight, body length, and tail length over a span of 4 weeks (initiating at Postnatal Day 1, PND1). Our findings revealed that all three parameters were significantly lower in CdCS rats compared to their WT littermates (*p* < 0.05 ≈ 0.0001, respectively, as illustrated in Figure 3B–D). We further analyzed the brain weight of 14‐week‐old CdCS rats and discovered that their brain weight was notably reduced compared to their WT littermates (Figure 3E, 1.63 ± 0.03 g for WT rat; 1.47 ± 0.05 g for CdCS rat; *p* < 0.01), aligning with the microcephaly phenotype commonly observed in CdCS patients.^[^ ^1^, ^14^ ^]^ These results strongly suggest that CdCS rats faithfully replicate the growth reduction observed in children with the 5p15.2 deletion in the study.

The genomic region 5p15.2 is thought to play a pivotal role in cerebral development, and its deletion has been implicated in cognitive impairments in individuals with CdCS.^[^ ^1^, ^3^, ^15^ ^]^ To gain insights into this phenomenon, we conducted a comprehensive assessment of nervous system development in CdCS rats. Our findings revealed that CdCS rats exhibited significantly delayed behavioral ontogeny compared to control rats, as evident in the prolonged postnatal days for the successful completion of both surface and air righting reflexes (Figure 3F). Intriguingly, our investigation into sensory functions disclosed an unexpected finding: the initial onset of auditory response in CdCS rats occurred markedly earlier than in their WT littermates (Figure 3G, left panel). However, no significant difference was observed in homing time of olfactory response between CdCS rats and their WT counterparts (Figure 3G, right panel). Further, CdCS rats demonstrated a notable decrease in forelimb grip strength and hanging endurance during the rope grip test (Figure 3H, left and right panels), which is consistent with the muscle hypotonia phenotype observed in CdCS patients.^[^ ^1^ ^]^ Collectively, these results suggest that CdCS rats exhibit a delayed progression of nervous system development, closely mirroring the clinical features seen in individuals with CdCS. In addition, our study showed that CdCS rats reached key developmental milestones such as eyelid opening and pinnae detachment, similar to controls (Figure 3I). Notably, no differences were detected in the negative geotaxis reflex (Figure 3F, right panel) between CdCS rats and WT rats. Similarly, no significant differences were observed in the stride lengths of both forelimbs and hindlimbs, as well as in stride width, between CdCS rats and WT rats (Figure 3J). This demonstrates regular gait activity, indicative of normal skeletal development and motor function. To further evaluate motor function and balance capacity, we conducted beam walking tests. Interestingly, CdCS rats exhibited normal balance capacity in the beam walking test, as evidenced by their ability to traverse the beam without falling (Figure 3K–M). These observations suggest that CdCS rats maintain normal cerebellar functions, which are vital for coordinating movement and preserving balance.

Taken together, our results indicate that while the 1.68 Mb chromosomal heterozygous deletion in 2q22 leads to delayed nervous system development and cognitive impairments in CdCS rats, it does not completely disrupt all aspects of brain development and function. This observation is consistent with clinical reports, which suggest that the severity of symptoms in CdCS patients is dependent on the size and location of the deleted region.^[^ ^1^ ^]^

### 2.3 Autistic‐Like Behaviors, Anxiety, and Cognitive Impairments

**Figure 4:**
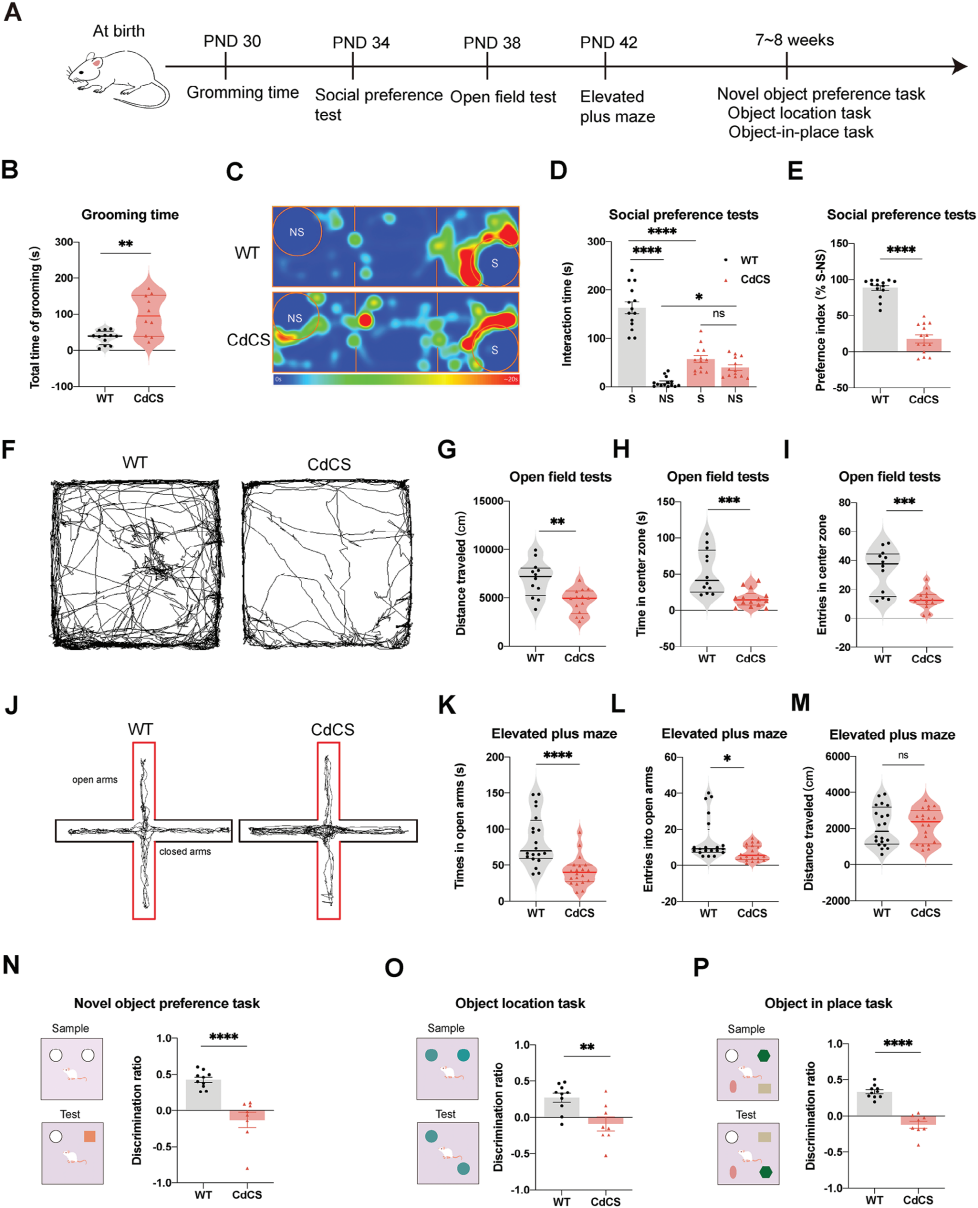
Altered social interaction and cognitive impairments in the CdCS rats. A) Overview of experimental procedures in WT and CdCS rats over time. B) CdCS rats groomed more than WT rats (WT: 35.67 ± 5.38 s vs CdCS: 98.00 ± 17.23 s, *p* = 0.0013). Sample sizes: WT = 12 (6F/6 M), CdCS = 10 (5F/5 M). C–E) CdCS rats showed social interaction defects in a social preference test (WT = 14, CdCS = 14). C) Representative trajectory data showing time spent in different chamber locations. D) CdCS rats spent less time with social stimulus (*p* < 0.0001). E) Lower social preference index in CdCS rats (WT: 88.31 ± 3.52% vs CdCS: 17.65 ± 5.36%, *p* < 0.0001). F–I) CdCS rats moved less in the open field test (OFT) (WT = 12, CdCS = 14). Representative traces (F). CdCS rats traveled shorter distances (WT: 6930.00 ± 535.40 cm vs CdCS: 4636.00 ± 339.40 cm, *p* = 0.0011) (G). CdCS rats spent less time in the center (WT: 53.32 ± 8.83 s vs CdCS: 16.61 ± 3.22 s, *p* = 0.0001) (H). CdCS rats visited the center less often (WT: 33.17 ± 4.34 times vs CdCS: 12.86 ± 1.74 times, *p* = 0.0001) (I). J–M) CdCS rats showed anxiety in the elevated plus maze (EPM) (WT = 20, CdCS = 20). Representative traces (J). CdCS rats spent less time in open arms (WT: 84.61 ± 7.95 s vs CdCS: 41.20 ± 4.49 s, *p* < 0.0001) (K). CdCS rats entered open arms less often (WT: 14.35 ± 2.65 times vs CdCS: 6.85 ± 0.96 times, *p* = 0.0210) (L). No difference in traveled distance (*P* = 0.9680). N–P) CdCS rats showed cognitive impairments (WT = 10, CdCS = 8) (M). N) Schematic diagram of novel object preference task (left panel). Reduced novel object preference in CdCS rats (WT: 0.42 ± 0.04 vs CdCS: −0.13 ± 0.11, *p* < 0.0001) (right panel). O) Schematic diagram of object location task (left panel). Impaired object location task in CdCS rats (WT: 0.27 ± 0.06 vs CdCS: −0.09 ± 0.10, *p* = 0.0049) (right panel). P) Schematic diagram of object‐in‐place task (left panel). Deficits in object‐in‐place task in CdCS rats (WT: 0.33 ± 0.03 vs CdCS: −0.12 ± 0.05, *p* < 0.0001) (right panel).

Individuals with CdCS exhibit features associated with ASD,^[^ ^5^ ^]^ and similar ASD‐like behaviors are also observed in *Ctnnd2* KO mice.^[^ ^5^, ^6^ ^]^ Given that heterozygous rats with the 2q22 deletion display significantly reduced gene expression, particularly *Ctnnd2*, our study aimed to investigate whether CdCS rats manifest some ASD‐like behavioral phenotypes. The hallmark features of ASD in humans encompass repetitive and stereotyped behaviors,^[^ ^16^ ^]^ and analogous behaviors can be observed and quantified in animal models of ASD.^[^ ^17^ ^]^ To address this aspect, we accurately assessed self‐grooming behaviors, encompassing licking or biting of paws or fur, in CdCS rats at PND 30 (**Figure** **4**A). Our results revealed that CdCS rats engaged in excessive grooming behaviors compared to WT rats (Figure 4B), suggestive of the presence of repetitive behaviors akin to those seen in ASD. Further, we conducted social preference test on CdCS rats at PND 34 to evaluate their sociability (Figure 4C). Notably, compared to control rats, CdCS rats spent less interaction time with the social stimulus (a rat) (Figure 4C,D). In addition, a reduced ratio of interactions with both stranger rat (social stimulus) and object (nonsocial stimulus) was evident in CdCS rats in contrast to WT rats (Figure 4E). Collectively, these findings indicate impaired sociability in CdCS rats relative to control rats. These observations converge to suggest that CdCS rats display behavioral deficits akin to core autistic behaviors observed in CdCS patients.

ASD is frequently accompanied by various comorbidities, including motor dysfunction,^[^ ^18^ ^]^ hyperactivity, and anxiety.^[^ ^19^ ^]^ As a result, we conducted assessments of locomotion and motor abilities on CdCS rats at PND38. Notably, CdCS rats walked considerably shorter distances than their WT counterparts in an open‐field test (Figure 4F,G), implying a hypoactivity trait rather than hyperactivity. Significantly, this hypoactivity trait does not closely mirror that observed in CdCS patients, thus distinguishing them from the typical ASD population^[^ ^20^ ^]^ and ASD mouse models.^[^ ^17^, ^21^ ^]^ Further, CdCS rats demonstrated a decrease in the duration spent and the number of entries into the central area of the open field (Figure 4H,I), as well as the open arms of the elevated plus maze (Figure 4J–L), without a notable change in their walking distance (Figure 3M). These findings are suggestive of anxiety‐like behaviors in these rats.

To ascertain the presence of cognitive impairments in CdCS rats, we conducted recognition memory assays encompassing the novel object preference task, object location task, and object‐in‐place task. Strikingly, CdCS rats demonstrated deficits in all three recognition memory tasks compared to WT rats (Figure 4N–P), mirroring the cognitive impairments frequently observed in CdCS patients. These results imply a dysfunction in the integrated neural network involving the hippocampus and cortical regions crucial for recognition memory.

Taken together, the cumulative findings from this study strongly indicate that CdCS rats exhibit ASD‐like behaviors, including impaired social interaction, and repetitive and stereotyped behaviors, along with cognitive dysfunctions, accompanied by comorbid hypoactivity.

### 2.4 Reduced Dendritic Complexity and Dendritic Spines, and Increased Neuron Density

Our observations of ASD‐like behaviors, impaired cognition, and hypoactivity in CdCS rats may reflect alterations in synapse formation and abundance. To probe the connection among delayed neural development, changed behaviors, and cognitive deficits in the brains of CdCS rats, we quantified dendritic arborization and dendritic spine formation across different brain regions during various developmental stages.

**Figure 5:**
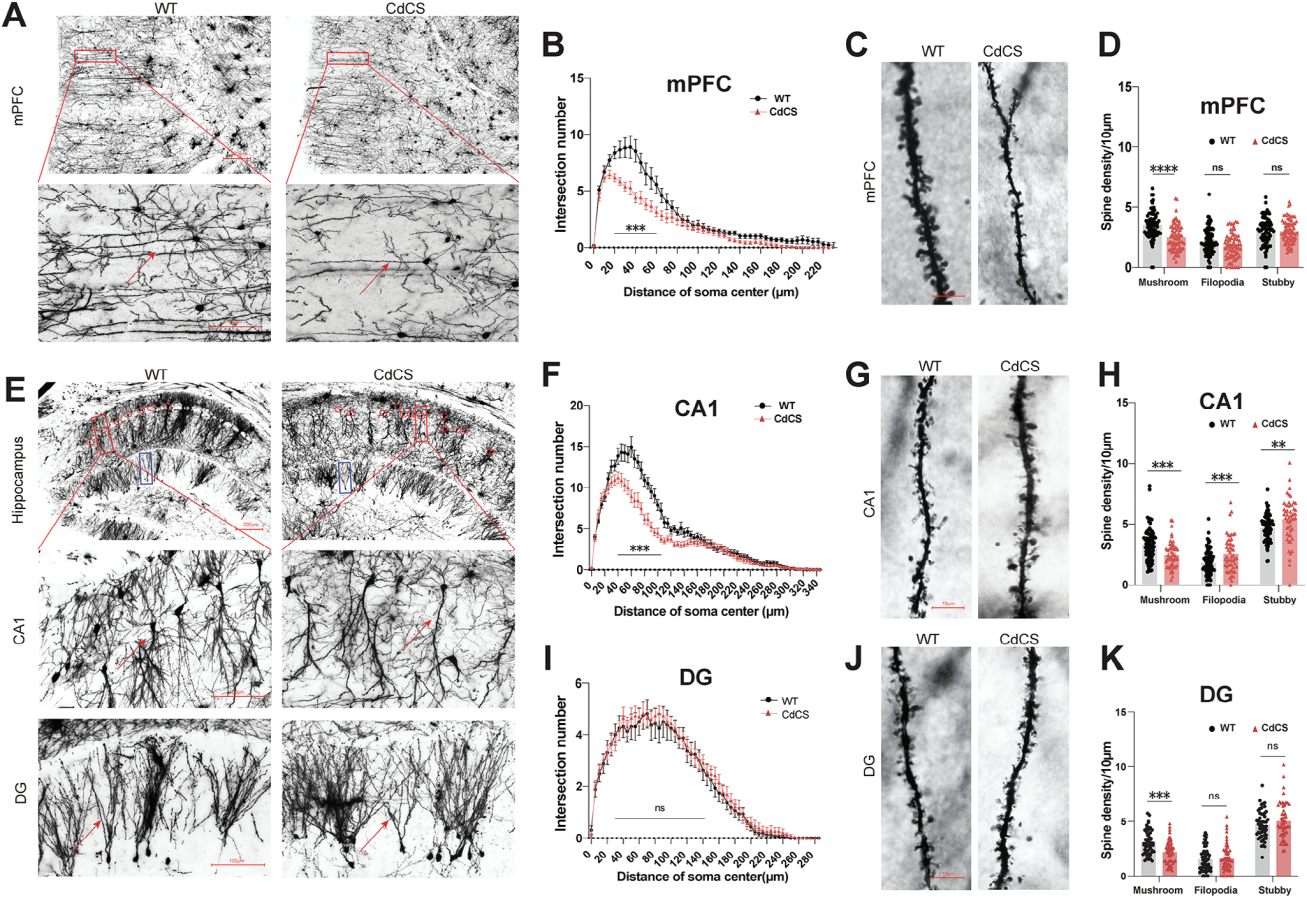
Impaired dendritic complexity and dendritic spines density in the CdCS rats. A) Golgi‐Cox‐stained neurons from the mPFC. Analyzed cells indicated by red box and arrow. Low‐mag: 200 µm scale bar. High‐mag: 100 µm scale bar. B) Sholl analysis shows decreased neuronal complexity in CdCS rats' mPFC versus WT. Less dendrites at 20–60 µm from soma (*p* < 0.001 for most distances). Sample sizes: WT = 18 cells/6 rats; CdCS = 24 cells/8 rats. C) Representative images of secondary dendrites in mPFC neurons. Scale bar: 10 µm. D) Significant reduction in mushroom‐like spines in CdCS rats' mPFC neurons (*p* < 0.001). No significant difference in fliopodia or stubby spines. Sample sizes: WT = 77 branches/8 rats; CdCS = 81 branches/8 rats. E) Golgi‐Cox‐stained neurons from hippocampus CA1 and DG regions. Analyzed cells indicated by red box and arrow. Low‐mag: 200 µm scale bar; High‐mag: 100 µm scale bar. F) Sholl analysis shows less dendrites in CdCS rats' CA1 region at 45–105 µm from soma (*p* < 0.001). Sample sizes: WT = 18 cells/6 rats; CdCS = 24 cells/8 rats. G) Representative images of secondary dendrites in CA1 neurons. H) Decreased mushroom‐like spines in CdCS rats' CA1 neurons (*p* = 0.0004). Increased fliopodia and stubby spines in CdCS (*p* < 0.001 for both). Sample sizes: WT = 67 branches/8 rats; CdCS = 63 branches/8 rats. I) No significant difference in neuronal complexity in DG region between CdCS and WT rats. Sample sizes: WT = 18 cells/6 rats; CdCS = 23 cells/8 rats. J,K) Decreased mushroom‐like spines in CdCS rats' DG neurons (*p* = 0.0001). No significant difference in fliopodia or stubby spines. Sample sizes: WT = 53 branches/8 rats; CdCS = 54 branches/8 rats. All data presented as mean ± SEM. Statistical significance: ***p < 0.001 and ****p < 0.0001.

Sholl analysis of Golgi‐Cox‐stained sections unveiled a significant reduction in dendritic arbor complexity within both the mPFC and the hippocampus CA1 of CdCS rats when compared to control rats (**Figures** **5**A,B,E,F, respectively). These differences emerged between 20 and 60 µm in the mPFC (Figure 5B) and between 40 and 105 µm in the hippocampal CA1 from the cell bodies (Figure 5F). Interestingly, no discrepancies in dendritic arbor complexity were found in the dentate gyrus (DG) regions of the hippocampus between CdCS and WT rats (Figure 5I). Our Golgi staining results also highlighted relatively disorganized neuron arrangement in the hippocampal CA1 region of the CdCS rats. In contrast, the control group displayed a comparatively smooth line of neuronal cell bodies (Figure 5E, upper and middle panels, marked by red circles).

Next, we conducted an in‐depth analysis of dendritic spine morphologies and density in the mPFC and hippocampus. We measured the density of spine protrusions in the secondary dendritic segments of layer II/III pyramidal neurons in the mPFC (Figure 5C), hippocampal pyramidal neurons in the stratum radiatum of the CA1 area (Figure 5G), and DG neuron dendrites (Figure 5J). Following established classifications, these spine protrusions were categorized as thin spines (those with a length‐to‐width ratio >2) or mushroom‐like spines.^[^ ^22^ ^]^ Given the absence of measurements for postsynaptic density and its presynaptic counterpart, we referred to these thin spine‐like structures as “filopodia” and “stubby.”^[^ ^23^ ^]^ Detailed quantitative analyses of layer II/III neuron dendrites in the mPFC uncovered a notable decrease in mushroom‐like spines (Figure 5D). Moreover, our observations revealed a persistent reduction in mushroom‐shaped spines within the dendrites of CA1 neurons in CdCS rats compared to WT counterparts, accompanied by a higher density of filopodia and stubby spines in CdCS rats than in WT rats (Figure 5H). Notably, a marked decrease in the density of mushroom‐shaped dendritic spines was observed in the dendrites of DG neurons from CdCS rats (Figure 5K). Conversely, no differences were detected in the overall density of filopodia and stubby spines among the dendritic spines of mPFC and DG neurons between CdCS and WT rats, respectively (Figure 5D,K).

Collectively, our data suggest that CdCS rats with the 2q22 deletion, leading to ≈50% decreased gene expression in most genes within the syntenic 5p15.2 region, exhibit deficits in spine density and maturation in the mPFC and hippocampus. Moreover, this deletion appears to contribute to the disorganized arrangement of neurons in the hippocampus.

Further, we employed fluorescence immunostaining assays to assess neuronal density in both the mPFC and hippocampus of 8‐week‐old CdCS rats (Figure S2A,B, Supporting Information). Surprisingly, we observed a significant increase in neuronal density within layer II/III (Figure S2C, left panel, Supporting Information) but not in layer V/VI of the mPFC, in CdCS rats when compared to WT rats (Figure S2C, right panel, Supporting Information). Neuronal density; however, remained unaltered in the hippocampus CA1 and DG regions in CdCS rats when compared to WT rats (Figure S2D, Supporting Information). This outcome suggests a pathophysiological increase in neuronal density occurring specifically within the mPFC superficial layer of CdCS rats during early adulthood.

### 2.5 Increase in Astrocyte Density and Complement C4 in the mPFC of CdCS Rats

Reduced dendritic complexity and alterations in dendritic spines are intricately regulated by astrocytes, microglia, and the complement cascade of the innate immune system, each contributing through diverse mechanisms. Astrocytes are pivotal in providing trophic support and modulating synaptic plasticity, fostering robust neuronal networks.^[^ ^24^ ^]^ However, they also engage in inflammatory processes that can potentially disrupt synaptic connections and impact dendritic spines under certain conditions.^[^ ^25^ ^]^ Conversely, microglia, the guardians of the brain's immune system, promptly respond to injury or disease by phagocytosing damaged neurons or synapses and releasing inflammatory signals,^[^ ^26^ ^]^ which may include the activation of the complement cascade, specifically involving complement C4.^[^ ^27^ ^]^ The intricate interplay between these cells and molecules ultimately dictates the preservation or deterioration of neuronal structure and function, thereby safeguarding the delicate balance of the central nervous system.

To explore the potential role of reactive astrocytes, microglia, and the complement cascade in inducing alterations in dendritic spines and decreased dendritic complexity in the brains of CdCS rats, we conducted a quantitative analysis of astrocyte and microglia densities, as well as complement C4 levels across distinct brain regions during various developmental stages (Figure S3, Supporting Information). Our findings revealed a significantly elevated astrocyte count in CdCS rats compared to WT rats, specifically in layer II/III of the mPFC (Figures S3A and S3C, Supporting Information, respectively), suggesting an inflammatory response localized to this layer. Conversely, no notable differences were observed in layer V/VI (Figure S3C, Supporting Information), indicating specificity to layer II/III. Intriguingly, no disparities in astrocyte density were detected between CdCS and WT rats in the hippocampal CA1 and DG regions (Figure S3D, Supporting Information).

Moreover, our comprehensive analysis failed to uncover any notable distinction in the quantity of Iba1‐positive (microglial) cells between CdCS and wild‐type (WT) rats across the scrutinized regions, spanning the mPFC layers II/III and V/VI, as well as the hippocampal CA1 and DG regions (Figure S3E,F, Supporting Information). Unexpectedly, an intriguing discovery emerged in the form of an elevated expression of complement C4c in the mPFC of CdCS rats compared to WT rats (Figure S3G,H). The elevated expression of complement C4c in CdCS rats indicates its activation state, offering valuable insights into the potential inflammatory pathways that could underlie the observed neuroanatomical changes in this CdCS rat model.

### 2.6 Gene Expression Landscapes in the mPFC and Hippocampus of CdCS Rats

**Figure 6:**
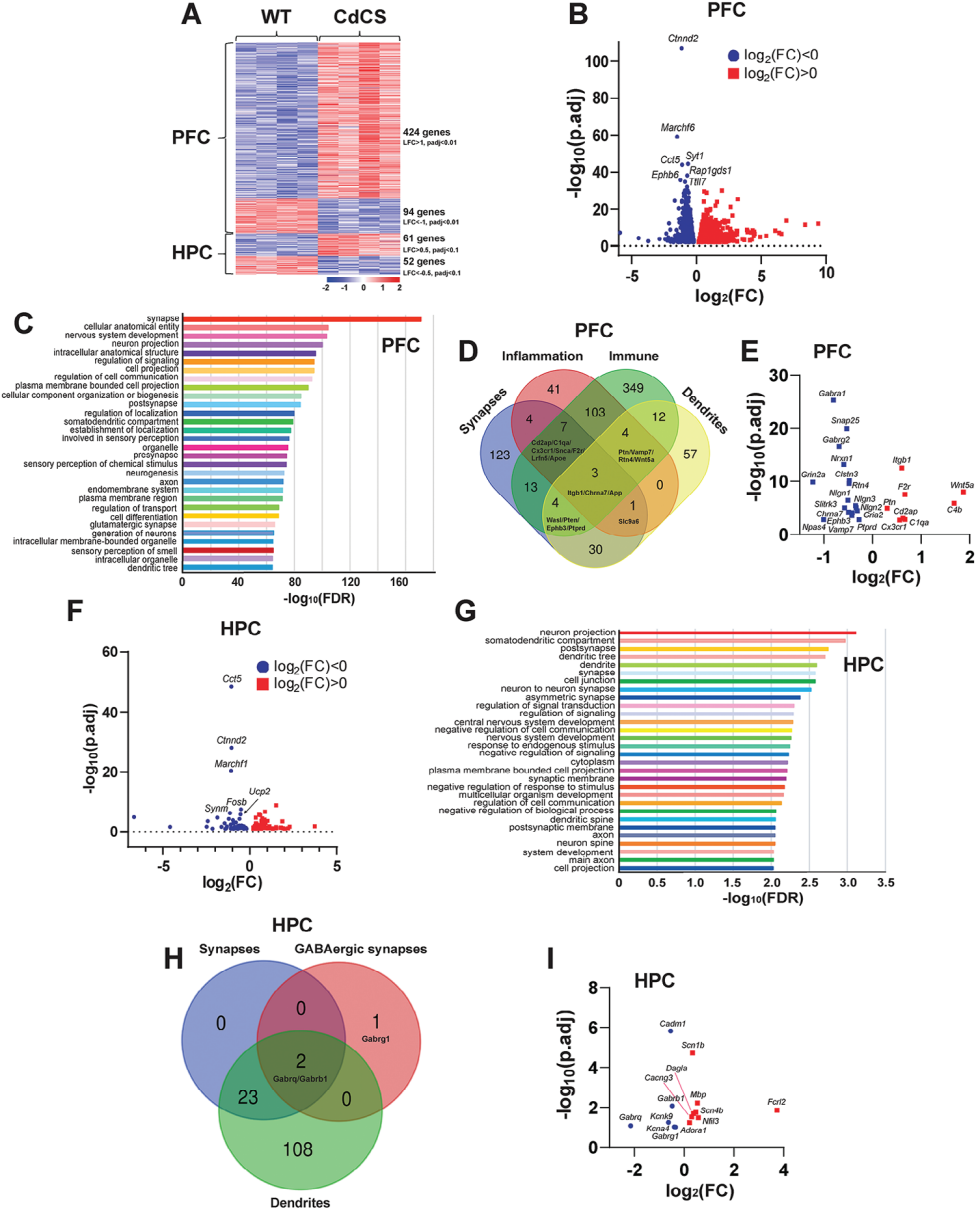
Altered gene expression patterns in the PFC and hippocampus of CdCS rats. A) A matrix heatmap visualization of genes exhibiting similar expression profiles in the PFC and hippocampus of CdCS rats compared to WT rats. B) Volcano plot of RNA‐seq differentially expressed genes (DEGs) in CdCS PFC (*p*.adj < 0.01). Red/blue dots signify upregulated/downregulated RNAs exceeding thresholds of log_2_FC > 0.22 and log_2_FC < −0.22, respectively. 3423 DEGs, including 1673 up and 1750 down, with significant downregulation of *Ctnnd2*, *Ankrd33b*, *Marchf6*, and *Cct5*. C) Gene set enrichment analysis (GSEA) of CdCS PFC DEGs enriched in synapse, nervous system development, neuron projection, and intracellular anatomical structure. D) Venn diagram of inflammatory and innate immune‐related DEGs in CdCS PFC. 163 inflammatory and 499 innate immune‐related DEGs. E) Fine‐tuned volcano plot of CdCS PFC DEGs. Key downregulation in synaptic/dendritic genes (*Nlgn1*, *Nrxn1*, *Nlgn2/3*, *Npas4*, *Clstn3*, *Ephb3*, *Slitrk3*, *Ptprd*, and *Rtn4*) and synaptic transmission/plasticity genes (*Snap25*, *Vamp7*, *Gabrg2*, *Gabra1*, *Grin2a*, *Gria2*, and *Chrna7*). Upregulation seen in inflammation/innate immune genes (*Cd2ap*, *C1qa*, *Cx3cr1*, *C4b*, *F2r, Ptn*, *Wnt5a*, and *Itgb1*). F) Volcano plot of 174 hippocampal DEGs in CdCS versus WT (*p*.adj < 0.1). 108 up and 66 down genes, with key downregulation of *Cct5*, *Ctnnd2*, and *Marchf6*. G) GO analysis of CdCS hippocampal DEGs enriched in neuronal structures, including neuron projection, somatodendritic compartment, postsynapse, dendritic tree, dendrite, and synapse, highlighting their importance in neuronal function and connectivity. H) Venn diagram of synaptic and dendritic alteration genes, specifically GABAergic synapses in CdCS hippocampus. I) Volcano plot of key hippocampal DEGs in CdCS: downregulation of *Gabrb1*, *Gabrg1*, *Gabrq*, *Kcna4*, *Kcnk9*, and *Cadm1* points to impaired GABAergic function, potassium channel activity, and synaptic adhesion. Conversely, upregulation of *Scn1b*, *Scn4b*, *Cacng3*, *Adora1*, *Mbp*, and *Dagla* hints at heightened neuronal excitability, AMPA receptor modulation, and myelin development, disrupting excitation/inhibition balance. Notably, *Nfil3* and *Fcrl2* upregulation signals inflammation and innate immune activation in the hippocampus.

To gain molecular insights into the impact of the heterozygous 2q22 deletion on nervous system development, especially regarding dendrite impairment and behavioral deficits during postnatal development, we performed RNA sequencing (RNA‐seq) on samples from the mPFC and hippocampus of 8‐week‐old WT and CdCS rats (Tables S1 and S2, Supporting Information). Through an analysis of differential gene expression profiles, we observed consistent patterns of gene expression between WT and CdCS rats in both the mPFC and HPC (**Figure** **6**A). This finding provides molecular evidence that the CdCS rat serves as a reliable and reproducible animal model for CdCS studies.

The analysis of differential expression profiling associated with the heterozygous 2q22 deletion uncovered a substantial set of 3423 differentially expressed genes, comprising 1673 upregulated genes and 1750 downregulated genes, between the mPFC regions of WT and CdCS rats (adjusted *p*‐value, *p*.adj <0.01; Figure 6B; Table S1, Supporting Information). The most significantly downregulated genes included *Ctnnd2* (log_2_FC = −1.13; *p*.adj = 9.50e‐112), *Ankrd33b* (log_2_FC = −1.67; *p*.adj = 2.94e‐25), *Marchf6* (log_2_FC = −1.48; *p*.adj = 8.35e‐64), and *Cct5* (log_2_FC = −1.10; *p*.adj = 1.40e‐44) (Figure 6B; Table S1, Supporting Information).

Gene set enrichment analyses demonstrated that these differentially expressed genes (DEGs) were mostly significantly enriched in “synapse” (FDR = 1.06e‐171, GO:0045202), “cellular anatomical entity” (FDR = 6.50e‐105, GO: 0110165), “nervous system development” (FDR = 8.67e‐104, GO: 0007399), “neuron projection” (FDR = 8.51e‐101, GO: 0043005), and “intracellular anatomical structure” (FDR = 6.97e‐96, GO: 0005622) (Figure 6C; Table S3, Supporting Information).

The inflammatory signals and the internal immune system are pivotal in the deterioration of brain synapses and dendrites. Aberrant activation of inflammatory signals can disrupt the plasticity and stability of synapses, culminating in cognitive impairment and the onset of mental disorders,^[^ ^28^ ^]^ as well as neurodegenerative diseases.^[^ ^29^ ^]^ Consequently, a rigorous exploration of the interplay between inflammatory signals and the internal immune system is imperative for elucidating the underlying mechanisms of CdCS and devising innovative therapeutic interventions. Thus, we performed a comprehensive analysis of inflammatory signaling pathways and the innate immune response within the PFC. Intriguingly, our findings identified 163 inflammatory and 499 innate immune‐related DEGs in the PFC of CdCS rats (Table S4, Supporting Information). To further elucidate the connection between these DEGs and synaptic/dendritic alterations, we conducted a gene intersection analysis, as shown in a Venn diagram (Figure 6D). This comprehensive analysis emphasized the pivotal role of inflammatory signaling pathways and innate immune responses in the pathogenesis of CdCS, establishing a connection between their DEGs and synaptic/dendritic alterations.

In addition, a conjoint examination of differential expression and gene ontology (GO) analyses indicated a pronounced downregulation of genes implicated in synaptic and dendritic development,^[^ ^30^ ^]^ notably *Nlgn1*, *Nrxn1*, *Nlgn2*, *Nlgn3*, *Npas4*, *Clstn3*, *Ephb3*, *Slitrk3*, *Ptprd*, and *Rtn4*, as well as those associated with synaptic transmission and plasticity,^[^ ^31^ ^]^ such as *Snap25*, *Vamp7*, *Gabrg2*, *Gabra1*, *Grin2a*, *Gria2*, and *Chrna7*, among the DEGs. Conversely, *Cd2ap*, *C1qa*, *Cx3cr1*, *C4b*, *F2r*, *Ptn*, *Wnt5a*, and *Itgb1*, genes linked to inflammation and innate immune responses in the brain,^[^ ^32^ ^]^ exhibited a significant upregulation in the PFC of CdCS rats (Figure 6E). To further validate our findings, we conducted qRT‐PCR analysis on independent PFC samples obtained from an additional cohort of WT and CdCS rats. This analysis confirmed a significant upregulation in the expression of genes related to inflammation and innate immune responses, specifically *C4b*, *F2r*, *Cd2ap*, and *Ptn* (*p < 0.05 and **p < 0.01; Figure S4 and Table S10, Supporting Information). These observations serve as a robust validation of our immunostaining data, reinforcing the notion that CdCS rats exhibit elevated C4 expression levels and inflammation within their brains. These findings imply that CdCS rats, characterized by a 2q22 deletion, undergo alterations in synaptic and dendritic development, which are accompanied by inflammatory and innate immune responses in the brain. It is crucial to highlight that C4b, a biologically active fragment, arises from the proteolytic cleavage of C4 during its activation phase. Analogously, C4c, which stems from the complement C4, signifies an additional fragment or manifestation that emerges concurrently with C4's activation. As C4b is inaccessible for animal studies, we utilized C4c as a trustworthy indicator to evaluate the activation status of C4 in immunostaining experiments.

Further, in contrast to PFC, our comprehensive RNA‐seq analysis of bulk tissue revealed only 174 DEGs, comprising 108 upregulated and 66 downregulated genes, between the hippocampi of WT and CdCS rats (adjusted *p*‐value, *p*.adj < 0.1; Figure 6F; Table S2, Supporting Information). Among the most significantly downregulated genes were *Cct5* (log_2_FC = −1.06; *p*.adj = 3.45e‐49), *Ctnnd2* (log_2_FC = −1.05; *p*.adj = 9.84e‐29), and *Marchf6* (log_2_FC = −1.08; *p*.adj = 4.38e‐21) (Figure 6F; Table S2, Supporting Information). The striking disparity in the number of DEGs between PFC and hippocampus in the context of 2q22 deletion remains largely unknown. This phenomenon could be attributed to the distinct temporal developmental windows and underlying mechanisms operative in these distinct brain regions.

The GO analyses conducted in the hippocampus revealed a significant enrichment of DEGs in various neuronal structures, including “neuron projection” (FDR = 7.66e‐4, GO: 0043005), “somatodendritic compartment” (FDR = 1.07e‐3, GO: 0036477), “postsynapse” (FDR = 1.79e‐3, GO: 0098794), “dendritic tree” (FDR = 1.97e‐3, GO: 0097447), “dendrite” (FDR = 2.54e‐3, GO: 0030425), and “synapse” (FDR = 2.65e‐3, GO: 0045202) (Figure 6G; Table S5, Supporting Information). Notably, these findings align with the results obtained from the PFC, reinforcing the observation that CdCS rats with a 2q22 deletion exhibit alterations in synaptic and dendritic development within the hippocampus.

Analogous to PFC analysis, we conducted an extensive gene intersection analysis of the hippocampus to clarify the relationship between these DEGs and synaptic/dendritic alterations in a Venn diagram (Figure 6H). This exhaustive analysis underscores the critical role of impaired synapses, specifically GABAergic synapses, and dendrites within the hippocampus, in the pathogenesis of CdCS, thereby establishing a link between their corresponding DEGs and synaptic/dendritic perturbations in this region.

Additional analysis revealed that the downregulation of specific DEGs, namely *Gabrb1*, *Gabrg1*, *Gabrq*, *Kcna4*, *Kcnk9*, and *Cadm1*, was intimately associated with diminished GABAergic function,^[^ ^31^, ^33^ ^]^ compromised neuronal voltage‐gated potassium channel activity,^[^ ^34^ ^]^ and reduced synaptic adhesion^[^ ^35^ ^]^ in the hippocampus (Figure 6I). Conversely, a noTable Supregulation was observed in genes such as *Scn1b*, *Scn4b*, *Cacng3*, *Adora1*, *Mbp*, and *Dagla*, which are intricately linked to neuronal voltage‐gated sodium channel function,^[^ ^36^ ^]^ modulation of AMPA receptor trafficking and gating,^[^ ^37^ ^]^ neuronal metabolism,^[^ ^38^ ^]^ and myelin development^[^ ^39^ ^]^ within the hippocampus (Figure 6I). This upregulation strongly suggests an imbalance in excitation/inhibition dynamics, particularly favoring increased neuronal excitability, within the hippocampal neural network. Remarkably, a significant upregulation was also detected in *Nfil3* and *Fcrl2* (Figure 6I), genes closely involved in innate immune responses,^[^ ^40^ ^]^ indicating the presence of inflammation and heightened innate immune activity within the hippocampus. Ultimately, this immune response further underscores the complex interplay between neuronal dysfunction and immune activation in CdCS.

### 2.7 A Single Injection of AAV‐*Ctnnd2* Effectively Rescued Cognitive Function and Modified Gene Expression Landscape; Yet, It Was Insufficient to Repair Sociability Dysfunction and Anxiety‐Like Behaviors

Given *CTNND2*’s extensive coverage in CdCS‐related deletions and its precise mimicry in rats, we hypothesized that an AAV‐PHP.eB vector expressing *Ctnnd2* could drive therapeutic CNS expression, thereby enhancing social behaviors and cognitive functions in CdCS rats with minimal immune response. AAV‐PHP.eB can penetrate the BBB and widely transduce CNS cells.^[^ ^12^, ^41^ ^]^ It is widely acknowledged that δ‐catenin plays a significant role in cancer, with its overexpression frequently observed in various cancers, including prostate (10.84%), liver (9.65%), breast (6.52%), ovary (6.02%), endometrium (5.56%), and lung (5.50%).^[^ ^42^ ^]^ In an effort to achieve overexpression of *Ctnnd2*; while, avoiding the risk of triggering cancer, we developed an AAV‐PHP.eB vector that carries a gain‐of‐function mutation of the *Ctnnd2* variant, incorporating core element domains akin to full‐length *Ctnnd2*, under the control of a CMV promoter. To evaluate its performance, we included EGFP for quantification across CNS cell types. Following tail vein intravenous injection of AAV‐*Ctnnd2* at a dosage of 5 × 10^11^ viral genomes per animal, robust CNS expression of the *Ctnnd2* variant and EGFP was confirmed after 4 weeks via cell transfection and immunofluorescence assays (Figure S5, Supporting Information).

**Figure 7:**
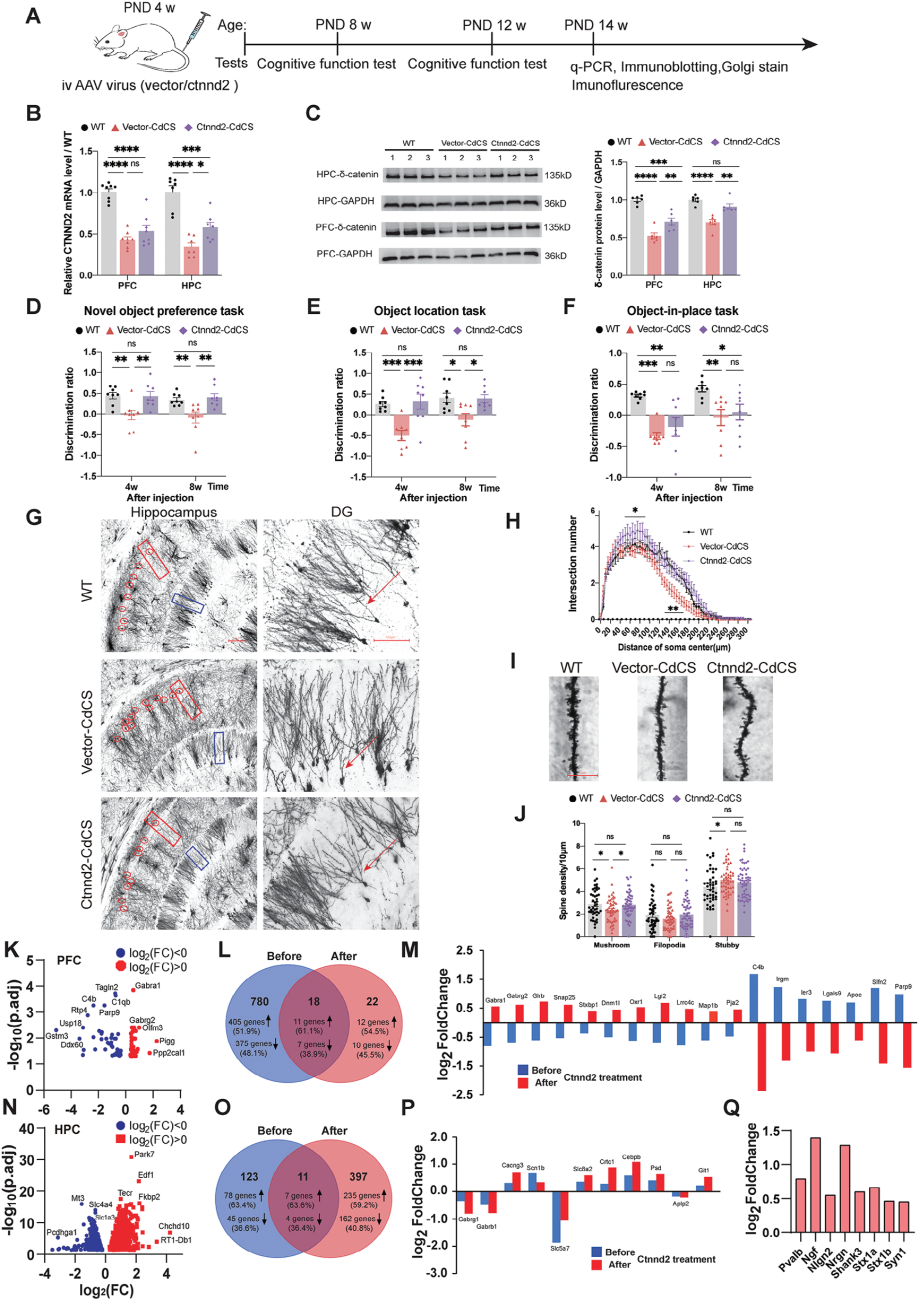
A single injection of AAV‐Ctnnd2 rescued the deficits of both social behavior and cognitive function at the early‐stage development in the CdCS rats. A) Timeline of tests performed on WT, Vector‐CdCS, and Ctnnd2‐CdCS rats. B) qPCR analysis showed elevated *Ctnnd2* mRNA levels in HPC of CdCS rats treated with AAV‐*Ctnnd2* compared to controls. Trends were noted in PFC but were not statistically significant. C) Immunoblotting analysis revealed increased δ‐catenin protein expression in PFC and HPC of rats administered AAV‐*Ctnnd2*. D–F) AAV‐*Ctnnd2* administration enhanced cognitive performance in CdCS rats in novel object preference task and object location tasks but not in the object‐in‐place task. G) Representative Golgi‐Cox‐stained neurons from hippocampal CA1 and DG regions. H) Dendrite analysis in DG showed more dendrites in Ctnnd2‐CdCS versus Vector‐CdCS rats at certain distances from the soma. I) Images of secondary dendrites in hippocampal DG of rats. J) In DG hippocampal neurons, mushroom‐like spine density was reduced in Vector‐CdCS rats but increased in Ctnnd2‐CdCS rats. No differences were observed in filopodia or stubby spines between groups. K,N) Volcano plots revealed distinct DEG patterns in PFC and hippocampus of CdCS rats post‐AAV‐*Ctnnd2* versus AAV‐EGFP. L) 40 PFC DEGs were identified, with 18 overlapping between pre‐ and post‐AAV‐*Ctnnd2* administration. M) AAV‐*Ctnnd2* administration in PFC reversed 11 downregulated genes from 18 DEGs, upregulating GABA_A_/glycine receptors, transmitter release, and synaptic/dendritic genes. Simultaneously, seven innate immune/inflammation genes downregulated, indicating suppression of immune response and inflammation. O) Substantial increase in hippocampal DEGs post‐AAV‐*Ctnnd2*, with limited overlap indicating treatment specificity. P) Among overlapping hippocampal DEGs, downregulation of GABA_A_ receptors and upregulation of voltage‐gated channels and synaptic genes were observed. Q) Upregulation of dendrite/synapse‐related genes in nonoverlapping set post‐AAV‐*Ctnnd2*, suggesting enhancement in neural processes despite limited rescue in overlapping DEGs.

As illustrated in **Figure** **7**A, the timeline depicts the tests conducted on WT, Vector‐CdCS, and Ctnnd2‐CdCS rats. Subsequently, we performed RT‐qPCR analysis to investigate whether intravenous administration of AAV‐PHP.eB‐CMV‐*Ctnnd2* led to an upregulation of the *Ctnnd2* gene expression. Notably, we observed a significant increase in the expression level of the *Ctnnd2* gene in the hippocampus (Figure 7B, right panel). While there appeared to be a trend toward enhanced expression in the PFC as well, no statistically significant difference was detected in this region (Figure 7B, left panel). Remarkably, the δ‐catenin, a coded protein by *Ctnnd2*, displayed a significant increase in the PFC and hippocampus of CdCS rats injected with AAV‐*Ctnnd2* compared to CdCS rats injected with AAV‐Vector (AAV‐EGFP); although, it remained lower than the expression in WT rats (Figure 7C).

Next, we examined the impact of AAV‐*Ctnnd2* administration on deficits in both social behavior and cognitive function across various developmental stages in CdCS rats. Initially, 4‐week‐old rats were intravenously injected with either AAV‐*Ctnnd2* or AAV‐Vector and underwent behavioral assays at 4 and 8 weeks post‐viral administration. The CdCS‐injecting AAV‐*Ctnnd2* (*Ctnnd2*‐CdCS) rats exhibited remarkable enhancement in the novel object preference task at both 4 and 8 weeks (Figure 7D). Notably, the administration of AAV‐*Ctnnd2* effectively restored the impaired object location memory at both time points (Figure 7E). However, despite this progress, the treatment with AAV‐*Ctnnd2* did not alleviate the object‐in‐place recognition deficit observed in CdCS rats (Figure 7F). Further, AAV‐*Ctnnd2* administration did not mitigate the sociability dysfunction, as evaluated at both the 4th and 8th week post‐injection in CdCS rats (Figure S6A,B, Supporting Information). In addition, it failed to rescue anxiety‐like behaviors in CdCS rats at both time points during the open‐field test (Figure S6C, Supporting Information).

Further, 8‐week‐old rats were also intravenously injected with either AAV‐*Ctnnd2* or AAV‐Vector, and behavioral assays were conducted at 4 weeks after viral administration. As shown in Figure S6D–G, Supporting Information, intravenous injection of AAV‐*Ctnnd2* did not alleviate the impaired recognition memories, including the novel object preference task, object location task, and object‐in‐place task after 4 weeks. Unfortunately, the recognition tasks mentioned above could not be performed due to significant hypoactivity observed in 16‐week‐old and older WT and CdCS rats.

To ascertain whether the enhancement of cognitive function in early‐stage CdCS rats administered with AAV‐*Ctnnd2* treatment is attributed to the improved dendritic and synaptic structures, we subsequently quantified dendritic arborization and spine formation. Our Golgi staining analysis revealed a notable augmentation in dendritic arbor complexity within the DG regions of the hippocampus in CdCS rats that received AAV‐*Ctnnd2* treatment, in contrast to CdCS rats administered with AAV‐Vector (Figure 7G,H). Further, we observed a sustained enhancement of mushroom‐shaped spines in DG neuron dendrites among CdCS rats treated with AAV‐*Ctnnd2* compared to those administered with AAV‐Vector (Figure 7I,J).

Our comprehensive investigation unveiled no statistically significant differences in neuronal complexity within the mPFC region among WT, Vector‐CdCS, and Ctnnd2‐CdCS rats (Figure S7A,B, Supporting Information). In the hippocampal CA1 region, CdCS rats treated with AAV‐Vector demonstrated a notable decrease in dendrites at distances ranging from 45 to 90 µm from the soma compared to WT rats. Nevertheless, no significant differences were detected between CdCS rats treated with AAV‐*Ctnnd2* and those administered AAV‐Vector across all distances examined (Figure S7C, Supporting Information). Notably, despite AAV‐Ctnnd2 treatment, the density of mushroom‐shaped spines remained decreased in the mPFC and hippocampal CA1 neuron dendrites of CdCS rats, indicating that the treatment did not fully restore this aspect of spine morphology. In addition, no notable differences were observed in the overall densities of filopodia and stubby spines across the dendritic spines of CA1 and DG neurons between CdCS rats treated with AAV‐*Ctnnd2* and those administered AAV‐Vector (Figure S7, Supporting Information; Figure 7J). However, an intriguing finding was that CdCS rats showed a pronounced reduction in the density of filopodia within the mPFC compared to Vector‐CdCS rats (Figures S7D, left panel, and S6E, Supporting Information). Notably, after AAV‐*Ctnnd2* treatment, CdCS rats exhibited a significantly higher density of filopodia compared to that of WT rats (Figure S7E, Supporting Information), suggesting a partial restoration of spine morphology toward normal levels specifically for filopodia.

In contrast to the observations in WT rats, the density of mushroom‐like spines was significantly reduced in both Vector‐CdCS and Ctnnd2‐CdCS rats within CA1 hippocampal neurons (Figure S7F, Supporting Information). However, no notable distinction was found in this regard between Vector‐CdCS and Ctnnd2‐CdCS rats (Figure S7F, Supporting Information). Regarding filopodia, no statistically significant differences were observed between WT rats and Vector‐CdCS rats, or between Vector‐CdCS rats and Ctnnd2‐CdCS rats. Intriguingly, the density of stubby spines was markedly increased in Vector‐CdCS rats compared to WT rats, whereas Ctnnd2‐CdCS rats did not show a significant deviation from Vector‐CdCS rats in this aspect (Figure S7F, Supporting Information).

Collectively, these findings indicate that a single injection of AAV‐*Ctnnd2* effectively elevates dendritic arbor complexity and the density of mature dendritic spines, implying that AAV‐*Ctnnd2* genuinely ameliorates neuronal and synaptic structures and functions.

In addition, we evaluated neuronal density within the mPFC 4 weeks after AAV‐*Ctnnd2* administration (Figure S8, Supporting Information). Unexpectedly, we failed to observe any significant alterations in neuronal density within layer II/III of the mPFC in CdCS rats that received AAV‐Vector injections compared to that of WT rats (Figure S8B, left panel, Supporting Information), in contrast to the notably increased neuronal density observed between CdCS and WT rats (Figure S2C, left panel, Supporting Information). This suggests that neurons within layer II/III of the mPFC in CdCS rats may undergo apoptosis or necroptosis, potentially triggered by inflammation and innate immune responses, ultimately leading to their demise. Notably, neuronal density in layer V/VI of the mPFC remained unchanged in CdCS rats following AAV‐*Ctnnd2* treatment (Figure S8B, right panel, Supporting Information). These findings imply that *Ctnnd2* treatment did not alter neuronal density in these regions, highlighting that this therapeutic approach does not have any toxic effect on neuronal loss.

To gain a deeper molecular understanding of how AAV‐*Ctnnd2* administration partially restores impaired recognition memories and modifies dendritic arborization, we further conducted RNA‐seq analysis on samples harvested from the mPFC and hippocampus of CdCS rats, 4 weeks post‐intravenous injection of AAV‐*Ctnnd2*. Remarkably, our analysis revealed a significantly lower number of DEGs in the PFC of CdCS rats, with 87 DEGs (comprising 48 upregulated and 39 downregulated genes) (Figure 7K), in contrast to a substantial increase in DEGs in the hippocampus, totaling 2394 DEGs (1230 upregulated and 1164 downregulated) (Figure 7N), following AAV‐*Ctnnd2* administration, as compared to intravenous injection of AAV‐EGFP (Tables S6 and S7, Supporting Information, respectively). Despite the lack of clarity regarding why the hippocampus exhibits a significantly higher number of DEGs compared to the PFC following AAV‐*Ctnnd2* administration, this observation appears to align with the notable upregulation of *Ctnnd2* gene and δ‐catenin expression levels in the hippocampus compared to the PFC (Figure 7B,C).

To investigate the complicated gene expression patterns associated with the pathogenesis of CdCS rats, we conducted an in‐depth RNA‐seq analysis, comparing pre‐ and post‐AAV‐*Ctnnd2* administration conditions. Our findings unveiled the presence of 40 DEGs, among which 18 were overlapping, in the PFC (Figure 7L; Table S8, Supporting Information). Upon the administration of AAV‐*Ctnnd2*, a remarkable reversal was observed in the PFC, with all 11 previously downregulated genes among the 18 DEGs experiencing complete upregulation (Figure 7M). This cohort of genes included two downregulated GABA_A_ receptor genes (*Gabra1* and *Gabrg2*), a downregulated glycine receptor gene (*Glrb*), and eight downregulated genes intricately linked to neuronal transmitter release and synaptic and dendritic development (*Snap25*, *Stxbp1*, *Dnm1l*, *Oxr1*, *Lgi2*, *Lrrc4c*, *Map1b*, and *Pja2*) (Figure 7M). This comprehensive upregulation implies the substantial restoration of GABAergic and glycine receptor functions, neuronal transmitter release, synaptic, and dendritic development in the PFC post‐AAV‐*Ctnnd2* injection. In contrast, the remaining seven upregulated genes among the 18 DEGs, known to be involved in innate immune response and inflammation,^[^ ^32^, ^43^ ^]^ underwent complete downregulation in the PFC (Figure 7M). These genes include *C4b*, *Irgm*, *Ier3*, *Lgals9*, *Apoe*, *Slfn2*, and *Parp9* (Figure 7M), pointing to a marked inhibition of innate immune response and inflammation in the PFC following AAV‐*Ctnnd2* administration.

In contrast to the only 40 DEGs identified in the PFC, a substantial increase of 408 DEGs was observed in the hippocampus (Figure 7O), when comparing pre‐ and post‐AAV‐*Ctnnd2* administration conditions. However, this significant change yielded only 11 overlapping DEGs (Figure 7O; Table S8, Supporting Information), highlighting the targeted specificity of the treatment's influence. Among these 11 DEGs, notable alterations included downregulation of two GABA_A_ receptor genes (*Gabrg1* and *Gabrb1*), upregulation of a calcium voltage‐gated channel auxiliary gene (*Cacng3*), a sodium voltage‐gated channel gene (*Scn1b*), and genes related to synaptic function and neural signaling, such as *Slc5a7* (choline uptake transporter), *Slc8a2* (cation transporter), *Crtc1* (CREB‐regulated transcription factor), *Cebpb* (CCAAT/enhancer binding protein), *Aplp2* (downregulated), and *Psd* and *Git1* (both upregulated) (Figure 7P). Unfortunately, none of these 11 overlapping DEGs exhibited rescue in the hippocampus subsequent to AAV‐*Ctnnd2* treatment (Figure 7P). Moreover, our findings illuminated the upregulation of 8 DEGs from the 397 nonoverlapping set (Figure 7O,Q; Table S8, Supporting Information), namely *Pvalb*, *Ngf*, *Nrgn*, *Shank3*, *Stx1a*, *Stxb1*, and *Syn1*, which were integral to dendrite and synapse development,^[^ ^44^ ^]^ synaptic transmission,^[^ ^45^ ^]^ and plasticity,^[^ ^45^, ^46^ ^]^ particularly in GABAergic function.^[^ ^44a^ ^]^ Notably, all eight genes were upregulated following AAV‐*Ctnnd2* treatment, suggesting an enhancement in these critical neural processes despite the lack of rescue in the above‐mentioned 11 overlapping DEGs. Thus, while complete restoration was not achieved for all affected genes, the observed upregulation underscores potential improvements in dendrite and synapse development, synaptic transmission, and plasticity within the hippocampus.

Further, we rigorously monitored the health status of the animals and procured blood samples for assessing liver and kidney function subsequent to vector infusion. Notably, we observed no mortality among the rats, and kidney function remained stable, maintaining normal levels for up to 8 months following AAV injection (Table S9, Supporting Information). However, it is worth noting that systemic AAV administration led to an elevation in total bilirubin levels in the blood serum (Table S9, Supporting Information). This observation suggests that the AAV‐PHP.eB capsid had a viral targeting affinity for liver cells, subsequently triggering an immune response and resulting in mild liver toxicity.

Upon administering AAV‐*Ctnnd2* via tail vein injection to mice, we recorded a complete absence of tumor incidence over a period of 1 year. This noteworthy observation underscores the non‐tumorigenic potential of AAV‐*Ctnnd2* in experimental models, thereby offering promising avenues for its therapeutic exploration in the treatment of CdCS.

Taken together, a single injection of AAV‐*Ctnnd2* effectively mitigated, to some extent, cognitive deficits during the early developmental stage, albeit not during adolescence and adulthood. Further, AAV‐*Ctnnd2* administration did not restore impaired sociability or anxiety‐like behaviors in CdCS rats.

## 3 Discussion

Cri du Chat Syndrome (CdCS), arising from a heterozygous deletion on the short arm of chromosome 5, stands as one of the most prevalent chromosomal deletion disorders in humans. Currently, there are no effective treatments available for CdCS.^[^ ^1^ ^]^ While there has been progress in comprehending specific pathogenic mechanisms, the development of effective disease‐modifying treatments for CdCS remains elusive. This challenge is rooted in several factors, with a significant obstacle being the lack of animal models that faithfully replicate the disease's pathogenesis; thus, hindering the evaluation of potential experimental treatments. This study aimed to establish an animal model that meticulously mirrors a prevalent genetic anomaly observed in CdCS patients and accurately reproduces the predominant pathogenic mechanisms driving CdCS. Another important aim was to advance a gene replacement therapy customized for the CdCS animal model and to explore a novel avenue for gene replacement therapy in Cri du Chat syndrome.

This study introduces a novel rat model for the 5p15.2 deletion syndrome, a prevalent genetic variation associated with Cri du Chat syndrome. Utilizing CRISPR‐Cas9 targeted engineering, we successfully generate a heterozygous deletion within the syntenic region on rat chromosome 2q22. Our findings reveal that rats harboring the 2q22 heterozygous deletion exhibit an ≈50% reduction in gene expression across critical genes within the syntenic 5p15.2 locus, including *Ctnnd2*, *Dap*, *Ankrd33b*, *Marchf6*, *Cmb1*, *Cct5*, and *Atpsckmt*. This comprehensive dataset underscores the fidelity of the 2q22 deletion rat model in faithfully recapitulating the genetic anomaly observed in the 5p15.2 deletion associated with Cri du Chat syndrome. Compared with some of the *Ctnnd2* KO mouse models,^[^ ^5^, ^6^ ^]^ our CdCS rats exhibit more comprehensive development, behavioral and cognitive deficits resembling humans with Cri du Chat syndrome. Specifically, the CdCS rats show remarkable growth deficits, delayed nervous system development, ASD‐like behaviors, including impaired social interaction, repetitive and stereotyped behaviors, along with cognitive dysfunction, accompanied by comorbid hypoactivity. Based on these results, we designate this model as the Cri du Chat syndrome rat model based on the 2q22 deletion. Leveraging this rat model of genetic deletion, which mirrors growth, behavioral, and cognitive deficits reminiscent of human Cri du Chat syndrome phenotypes, provides a valuable platform for in‐depth exploration of the pathophysiology and potential therapeutic interventions for Cri du Chat syndrome.

Moreover, our study uncovers key neuronal phenotypes in CdCS rats, notably reduced dendritic arbor complexity and mature spines in the hippocampus and mPFC. These synaptic alterations mirror those seen in *Ctnnd2* KO mice, suggesting a crucial role in CdCS etiology. In addition, our novel CdCS rat model exhibits increased neuronal density and disrupted arrangement in the mPFC and hippocampus, not observed in previous mouse models,^[^ ^5^, ^6^ ^]^ emphasizing Ctnnd2's significance in synaptic development. The involvement of other genes, including *Dap*, *Ankrd33b*, *Ropn1l*, *Marchf6*, *Cmbl*, *Cct5*, and *Atpsckmt* in the 5p15.2 region, underscores their roles in brain development and neuropathologies.^[^ ^47^ ^]^ To the best of our knowledge, our study presents convincing evidence of the remarkable fidelity of the 2q22 deletion rat model in accurately mirroring the genetic aberration corresponding to the 5p15.2 deletion. This model has significantly contributed to elucidating the intricate interplay among delayed neural development, behavioral alterations, and cognitive impairments observed in the brains of CdCS rats, which are analogous to those found in Cri du Chat syndrome.

δ‐Catenin, encoded by *CTNND2*, belongs to the adhesive junction‐associated protein armadillo/beta‐catenin superfamily, uniquely expressed within the nervous system.^[^ ^48^ ^]^ Most pronounced in the fetal brain, alterations in this gene relate to neurodevelopmental disorders.^[^ ^5^, ^49^ ^]^ In Cri du Chat syndrome, *CTNND2* deletion—common with large chromosome 5p deletions—yields severe neurological symptoms.^[^ ^1^, ^15^, ^50^ ^]^ Genetic variations in *CTNND2*, including structural and single nucleotide variants, associate with conditions such as ASD,^[^ ^5^, ^51^ ^]^ anxiety,^[^ ^52^ ^]^ schizophrenia,^[^ ^53^ ^]^ intellectual disability,^[^ ^15a,b^ ^]^ neurodevelopmental delay,^[^ ^5g^ ^]^ epilepsy,^[^ ^54^ ^]^ cerebral palsy,^[^ ^55^ ^]^ attention deficit hyperactivity disorder (ADHD),^[^ ^56^ ^]^ and depression.^[^ ^57^ ^]^ *Ctnnd2* influences neuronal differentiation,^[^ ^58^ ^]^ dendrite/synapse functions,^[^ ^3^, ^5^, ^54^, ^59^ ^]^ and neurogenesis,^[^ ^49^ ^]^ regulated via actin cytoskeleton interaction, ubiquitin proteasome pathway, and autophagy.^[^ ^58^, ^59^, ^60^ ^]^ *CTNND2* has been demonstrated to interact with a range of synaptic molecules, including N‐Cadherin,^[^ ^59^, ^61^ ^]^ Presenilin,^[^ ^62^ ^]^ RhoGTPases,^[^ ^42^, ^63^ ^]^ and Shank3.^[^ ^64^ ^]^ In mice, *Ctnnd2* loss reduces learning and induces ASD‐like traits such as sociability decline and heightened anxiety.^[^ ^3^, ^7^ ^]^ Conversely, *Ctnnd2* overexpression enhances recognition, sociability, and reduces anxiety.^[^ ^65^ ^]^

A recent study emphasizes the crucial role of δ‐catenin in astrocytes, which is indispensable for orchestrating astrocyte morphogenesis through the cadherin–catenin complex.^[^ ^66^ ^]^ It acts as a mediator in the interactions between astrocyte–neuron cadherins,^[^ ^66^ ^]^ thereby shaping astrocyte morphology and suggesting its involvement in neurogenesis. Our CdCS rats with *Ctnnd2* deletion exhibit behaviors reminiscent of ASD, cognitive deficits, and anxiety, reinforcing the hypothesis that the loss of function of *Ctnnd2* underlies these phenotypic manifestations. This observation underscores the intricate involvement of δ‐Catenin in the nervous system and underscores the pivotal role of astrocytes in synapse formation. Consequently, future research endeavors should prioritize these aspects for deeper exploration.

Genetic modifications have long been advocated for treating hereditary disorders, offering lasting clinical benefits.^[^ ^10^ ^]^ Adeno‐associated virus (AAV)‐based gene therapy is a promising strategy, with approved treatments such as Luxturna, Zolgensma, and CAR‐T therapies.^[^ ^10^ ^]^ Clinical trials are ongoing for Hemophilia B and β‐Thalassemia treatments.^[^ ^11^ ^]^ AAV‐PHP.eB's efficiency in brain transduction underscores its potential for addressing neurological disorders.^[^ ^10^, ^12^ ^]^ Given the crucial role of *CTNND2* in CdCS and the need to mitigate the risk of cancer induction,^[^ ^42^ ^]^ we developed AAV‐PHP.eB, carrying a gain‐of‐function *Ctnnd2* variant, placed under a CMV promoter for wide brain distribution. Early‐stage intravenous AAV‐*Ctnnd2* injection significantly boosts δ‐catenin in the mPFC and hippocampus, notably rescuing cognitive deficits in CdCS rats. However, AAV‐*Ctnnd2* does not restore sociability or anxiety‐like behaviors. Administration of AAV‐*Ctnnd2* to adolescent/adult CdCS rats also fails to reverse deficits.

In addition, systemic AAV administration increases in total bilirubin levels in blood plasma, indicating the AAV‐PHP.eB capsid's affinity for liver cells and mild liver toxicity. These findings highlight three aspects:1) AAV‐*Ctnnd2* therapy's effectiveness relies on optimal time windows, favoring earlier interventions. This insight has implications for treating CdCS patients. 2) The CdCS rat model encompasses the deletion not only of *Ctnnd2* but also of seven other genes within the syntenic 5p15.2 region—*Dap*, *Ankrd33b*, *Ropn1l*, *Marchf6*, *Cmbl*, *Cct5*, and *Atpsckmt*. While the specific functions of these genes in the central nervous system remain largely unclear, various studies have suggested that these genes likely contribute to nervous system development and neuropathologies.^[^ ^47^ ^]^ Thus, solely using *Ctnnd2* gene replacement is insufficient to fully rescue CdCS phenotypes, indicating the potential necessity for undertaking multiple gene replacements and adopting complementary strategies, such as the use of chemical compounds to enhance the expression of one or more of these genes associated with brain development and neurogenesis. 3) The AAV‐PHP.eB capsid's liver cell targeting affinity and mild toxicity underscore the necessity of developing novel AAV capsids with minimal liver expression, such as AAV.CAP‐B10,^[^ ^67^ ^]^ in the near future.

Our immunostaining experiment and RNA‐seq analysis of the CdCS rat have shed light on a novel aspect of the disease, uncovering the presence of inflammation‐ and innate immunity‐related responses in key brain regions. Notably, systemic AAV‐*Ctnnd2* administration effectively attenuates innate immune response and inflammation in the PFC, reinforcing the pivotal role of these pathways and hinting at a potential therapeutic breakthrough. A comprehensive approach that combines anti‐inflammatory interventions with gene replacement therapies holds great promise for tackling CdCS, with significant implications for advancing both research and treatment options for this complex syndrome.

Moreover, contrasting PFC, our RNA‐seq data reveals a significantly smaller number of DEGs in the hippocampus versus the PFC in the context of 2q22 deletion. The pronounced disparity in the number of DEGs between these two brain regions remains largely enigmatic. This phenomenon might stem from the unique temporal developmental trajectories and underlying mechanisms that govern these distinct brain regions. Surprisingly, administering AAV‐*Ctnnd2* to early‐stage CdCS rats elicits a substantially higher number of DEGs in the hippocampus compared to the PFC. This molecular mechanism remains a puzzle and warrants further investigation. Our findings suggest that following AAV‐*Ctnnd2* administration, there is a markedly higher expression level of the *Ctnnd2* gene and δ‐Catenin protein in the hippocampus than in the PFC, suggesting that increased exogenous *Ctnnd2* expression drives a corresponding increase in DEGs. If this is indeed the case, it implies that augmenting *Ctnnd2* gene expression may lead to enhanced therapeutic outcomes, which necessitates exploration in future studies.

Our investigation revealed a pathological increase in neuronal density in the superficial layer of the mPFC in CdCS rats. Conversely, AAV‐Vector administration as a control showed no alterations in neuronal density, indicating age‐dependent apoptosis/necroptosis, possibly orchestrated by unique inflammation and immune responses. Notably, AAV‐*Ctnnd2* intervention did not modify neuronal density, suggesting that despite δ‐Catenin's involvement in neuronal development and plasticity, its overexpression had no short‐term impact on neuronal count or density. This observation highlights the intricate role of δ‐Catenin in the nervous system and demonstrates no toxic effects on neuronal loss. It implies that δ‐Catenin's effects influence neuronal morphology, functional connectivity, synaptic plasticity, and ultimately, animal behavior and cognitive performance. Therefore, future investigations should focus on these aspects.

The brain controls various functions, including cognition, anxiety, and social behavior. The PFC manages higher cognitive tasks;^[^ ^28c^ ^]^ while, the hippocampus regulates emotions and social interactions, impacting anxiety^[^ ^68^ ^]^ and social behavior.^[^ ^69^ ^]^ Our study, using RNA‐seq data, found significant gene alterations in both the PFC and hippocampus, especially in the PFC, in the presence of a specific chromosomal deletion at 2q22. After administering AAV‐*Ctnnd2*, we observed an increase in *Ctnnd2* gene expression in the hippocampus, but only slightly in the PFC. The hippocampus showed more gene changes after gene therapy, which correlated with higher expression of *Ctnnd2* and δ‐catenin. Notably, gene therapy rescued overlapping gene changes in the PFC but not in the hippocampus. These differences in gene expression may explain why cognitive function improved but not anxiety and social behavior after AAV‐*Ctnnd2* treatment, potentially due to varying degrees of synaptic and dendritic repair.

Based on general understanding, we hypothesize that the utilization of the full‐length *Ctnnd2* gene would be more efficacious in enhancing brain function compared to a gain‐of‐function mutated variant. However, achieving overexpression and augmenting the activity of *Ctnnd2* presents a cancer risk as δ‐catenin plays a crucial role in cancer, and its overexpression is commonly observed in various types of cancer.^[^ ^42^ ^]^ A recent study has confirmed that gene therapy aimed at halting Cerebral Adrenoleukodystrophy, a brain disease, can also induce blood cancer, potentially due to the virus used to deliver the therapeutic gene.^[^ ^70^ ^]^ Given this cancer risk, we designed and implemented a gain‐of‐function mutated *Ctnnd2* variant, acknowledging that it might result in inferior brain function compared to full‐length *Ctnnd2* gene therapy. Considering that peripheral cells may exhibit higher overexpression of *Ctnnd2* than brain cells due to the blood–brain barrier, there is a heightened risk of tumorigenesis. Following the administration of this modified AAV‐*Ctnnd2* via tail vein injection to rats, we observed no incidence of tumors over a 1 year period. We consider this aspect of our study to be novel and significant as it underscores the non‐tumorigenic potential of AAV‐*Ctnnd2* in laboratory models, suggesting promising therapeutic avenues for the treatment of CdCS.

Recent advancements in non‐viral CRISPR/Cas9 nanoformulations have opened new doors for gene therapy due to their reduced immunogenicity and enhanced safety.^[^ ^71^ ^]^ However, these nanoformulations often lag behind AAV vectors in delivery efficiency, particularly when targeting the central nervous system due to difficulties in crossing the blood–brain barrier.^[^ ^72^ ^]^ In contrast, AAV vectors have demonstrated effectiveness in crossing this barrier and delivering genes to specific cells, with the capacity to sustain long‐term gene expression.^[^ ^41^, ^67^, ^73^ ^]^ Both methods have their respective advantages and disadvantages in treating brain diseases. Non‐viral CRISPR/Cas9 nanoformulations offer superior safety but encounter challenges in delivery, stability, and cytotoxicity.^[^ ^71^ ^]^ Conversely, AAV‐mediated gene therapy excels in delivery efficiency, targeted cell specificity, and sustained gene expression but may elicit immune responses, have limited payload capacity, and be susceptible to neutralizing antibodies.^[^ ^41^, ^67^, ^73^ ^]^ Ultimately, the choice between these two methods will hinge on the specific therapeutic requirements, target cell type, and other pertinent factors.

In conclusion, we have successfully developed a precise CdCS rat model that accurately reflects a prevalent genetic anomaly, replicating crucial pathogenic mechanisms and revealing the presence of inflammation and innate immunity, along with novel alterations in gene expression within these pathways in the PFC and hippocampus. This underscores their pivotal role in CdCS pathogenesis and paves the way for innovative therapeutic strategies, particularly, tailored gene replacement therapies aimed at early intervention.

## 4 Experimental Section

### Animals

Wild‐type SD (Sprague Dawley) rats and CdCS rats were bred in the animal facility of the Chongqing Medical University. All rat experiments were carried out according to the recommendations of AAALAC (Association for Assessment and Accreditation of Laboratory Animal Care International). The IACUC (Institutional Animal Care and Use Committee) of Chongqing Medical University approved the animal protocol (15‐LB5) used in this study. All efforts were made to minimize animal suffering during the experiments. Rats were maintained on a standard 12 h light/12 h dark cycle and were housed in groups of one to two. Food and water were provided ad libitum at constant room temperature (24 °C ± 2 °C). The experiments were performed in both male and female rats.

### Generation of 5p15.2 Deletion (CsCS) on SD Rats

To generate the 5p15.2 deletion in the rat, two CRISPR gRNAs were designed at syntenic loci in the rat genome: ATGTCGATGTCTTGTTAGGGTGG at Chr.2:83120000 (RGSC 6.0/rn6) and GCTGAGATGGCTTTCAGAAATGG at Chr.2:84800000 (RGSC 6.0/rn6), which induced ≈1.68 Mb chromosomal deletion. The Biocytogen Transgenic and Gene Targeting core injected 50 ng uL^−1^ of each gRNAs and 100 ng uL^−1^ Cas9 RNA into single‐cell SD rat zygotes. Embryos were cultured overnight and transferred to pseudopregnant females. PCR was used to screen for the deletion. PCR was performed using genomic ear or tail DNA, and the following primer pair: Proximal Forward‐TTGCTCAGCTGTTAAGGGAAACTAT and Distal Reverse‐TCATCAAAATGCACCAAAAGTGCAA (these primers generate ≈485 bp product). PCR primers were used to detect the breakpoint on the undeleted 5p15.2 interval (wild‐type allele): Proximal Forward‐TTGCTCAGCTGTTAAGGGAAACTAT and Proximal Reverse‐GGAGTAAGTCAACTGACTAGGGGACA (these primers generate ≈618 bp product).

PCR conditions were as follows: 94 °C for 3 min, 32 cycles at 94 °C for 15 s, 62 °C for 20 s, and 72 °C for 60 s; then, one cycle at 72 °C for 7 min. F1 rats were positively confirmed by PCR product sequencing (Figure S1, Supporting Information).

### Golgi‐Cox Analysis

Golgi‐Cox staining was performed to examine the dendritic arbor complexity and neuronal spine density of the mPFC and hippocampus using Golgi‐Cox OptimStainTM PreKit (Hitobiotec Corp, TN, USA). According to the manufacturer's instructions, after deep anesthetisia, the brains of rats were rapidly removed. Followed rinsing in distilled water, brains were immersed in a 1:1 v/vmixture of Solutions 1 and 2 for 2 days, and then, kept at room temperature for 2 weeks. Brains were then immersed in Solution 3 and stored at 4 °C for another 3 days. The brains used for Golgi‐Cox staining were sectioned on a vibratome in the sagittal plane at 100 µm. Slides were processed for Golgi stained directed by the manufacturer after drying in the dark at room temperature. The neuronal cells in the mPFC or hippocampal CA1 were imaged using a bright‐field microscope (Leica, Germany). Three neurons from each region per rat were randomly selected for sholl analysis and three to five neurons per rat were analysis of spine density using the ImageJ analysis system. For each neuron, the number of spines was counted within 20–100 µm segments on the secondary dendrites. Images were inverted and processed, segmented, and detected using ImageJ software.

### Histology

Rats were deeply anesthetized with an overdose of chloral hydrate and perfused transcardially with 250 mL of 0.9% saline, followed by 250 mL of 4% paraformaldehyde in phosphate‐buffered saline (PBS). Brains were removed, postfixed in 4% paraformaldehyde solution for 24 h, and dehydrated in increasing concentrations (10%, 20%, and 30%) of sucrose solution for 3 days at 4 °C. Thereafter, frozen medial prefrontal cortex (mPFC) and hippocampus tissues were serially sectioned on a cryostat into 30 µm coronal sections, they were then collected into six‐well plates containing cryoprotectant solution (30% ethylene glycol and 20% glycerol in PBS), and five series were collected and stored; one of the six series was analyzed by each of the staining methods.

### Cell Counting and Fluorescent Intensity Detect

Immunohistochemical staining was performed with NeuN (neuronal marker). Slices were removed from the cryoprotectant solution, rinsed three times in phosphate‐buffered saline (PBS, 0.01 m), and incubated for 60 min with 1% Triton X‐100 in PBS to permeabilize the cell membrane. The tissue was then incubated with blocking solution (5% BSA, 0.3% Triton X‐100 in PBS) for 1 h to decrease non‐specific labeling. Sections were incubated with mouse anti‐neuronal nuclei (NeuN) antibody (1:300; Abcam, USA), anti‐GFAP (1:200; Boster, China), anti‐IBA‐1(1:200; Boster, China), and anti‐C4c (1:100; ABclonal, China) at 4 °C for 48 h. Then was PBS washing, followed by Alexa Fluor 488‐labeled goat anti‐mouse IgG, Alexa Fluor 549‐labeled goat anti‐mouse IgG, Alexa Fluor 549‐labeled goat anti‐rabbit IgG, Alexa Fluor 488‐labeled goat anti‐rabbit IgG (1:200, Beyotime Institute of Biotechnology, China), and secondary antibody for 60 min at room temperature. at 4 °C for 48 h. Washing with PBS was followed by Alexa Fluor 488‐labeled goat anti‐mouse IgG (1:200, Beyotime Institute of Biotechnology, China) secondary antibody for 60 min at room temperature. Samples were incubated in 496‐diamidino‐ 2‐phenylindole (DAPI) (Sigma–Aldrich, USA) for 2 min to counterstain nuclei. Digitally captured images were captured using Thunder Imager and Leica LAS X Core imaging software (Leica, Germany). Morphometric analysis was performed by cell number quantification (NeuN) in every five section (150 µm separation distance, six sections per animal) in the mPFC and CA1, DG subfields of the hippocampus.

Cell counting was performed by cell number quantification (NeuN, GFAP, IBA‐1) in every three to five sections (150 µm separation distance, five sections per animal) in the mPFC and CA1, DG subfields of the hippocampus. In each section of the anti‐NeuN, anti‐GFAP, and anti‐IBA‐1, positive neurons were expressed as the number of neurons per 10^5^ µm^−3^ of each region analyzed by using Imaris software (Bitplane Inc., Zurich, Switzerland). The fluorescent intensity of C4c was calculated by using LAS X imaging software (Leica, Germany).

### Viral Vector Construction and Preparation

Rat *Ctnnd2* isoform 1 (NM_001271502.2) of 2079 bp (1663‐3741) was cloned into the EcoRI (689)/AgeI (1578) site of the pAAV‐CMV‐EGFP‐3xFLAG‐tWPA (H9525, OBIO, Shanghai) to create pAAV‐CMV‐rCTNND2‐P2A‐EGFP‐tWPA construct, for which correctness of the construct was confirmed by sequence measurement. Plasmids carrying the transgene cassette flanked by viral inverted terminal repeats (ITRs) (sspAAV‐PHP.eB‐CMV‐rCTNND2‐P2A‐EGFP‐tWPA), a rep‐cap expression construct encoding the sequence for the AAV‐PHP.eB serotype capsid, and a helper plasmid expressing adenoviral E2a, VA, and E4‐orf6; were transfected into mammalian HEK293 producer cells by Lipofectamine 3000 in DMEM media supplemented with 10% FBS. After 3 days, cells were collected and AAVs were purified by AAVpro Purification Kit of Clontech following the manufacturer's instruction. Quality control was performed to ensure purity by viral capsid protein evaluation by silver staining on SDS‐PAGE. Viral titers were determined by qPCR of purified vector particles using a CMV primer‐probe set: forward: 5′‐ CGCAAATGGGCGGTAGGCGTG‐3′, reverse:5′ – AGAGACAGCAACCAGGAT‐3′.

### Brain Region‐Specific RNA‐Seq and Bioinformatics Analyses

The PFC and hippocampus regions from 9 and 14‐week‐old rats were isolated and homogenized using TissueLyser LT (Qiagen, Germany). Total RNA was utilized as the starting material for RNA sample preparation. mRNA was purified from total RNA via poly‐T oligo‐attached magnetic beads, fragmented using divalent cations in first strand synthesis reaction buffer(5X), and then synthesized into first‐strand cDNA with random hexamer primer and M‐MuLV Reverse Transcriptase(RNase H‐). Subsequent second‐strand cDNA synthesis was catalyzed by DNA Polymerase I and RNase H. Overhangs were converted to blunt ends, and adaptors with hairpin loops were ligated to 3′ adenylated DNA fragments. The library fragments, selectively 370–420 bp in length, were purified with AMPure XP and amplified by PCR with Phusion High‐Fidelity DNA polymerase and primers. PCR products were purified and quality‐checked on an Agilent Bioanalyzer 2100. Indexed samples were clustered on a cBot with TruSeq PE Cluster Kit v3‐cBot‐HS (Illumina) and sequenced on an Illumina Novaseq platform, generating 150 bp paired‐end reads. Reads were aligned to the RGSC 6.0/rn6 rat genome using Hisat2 v2.0.5, guided by Ensembl GTF, and gene expression was quantified with featureCounts.

### Statistics

Statistical analysis was performed by using Prism 8.0 software (GraphPad Software). Experiments with two groups were analyzed using the Shapiro–Wilk test for normality and lognormality test, *α* = 0.05. If the result conformed to the normality, unpaired parametric two‐tailed *t*‐tests were used; otherwise, the Mann–Whitney test was performed. Experiments with more than two groups were subjected to one‐way or two‐way ANOVA followed by Tukey's multiple comparison test. The results were considered significant at < 0.05. Data are presented as mean ± SEM.

## Conflict of Interest

The authors declare no conflict of interest.

## Author Contributions

F.Y. and S.W designed the CdCS rat model; J.S., Y.W., S.L, and J.L. performed the biochemical assays, analyzed the data; J.S., Y.W., S.L., J.L., Y.B.L, D.C., and J.Z. conducted and engaged in the behavioral analysis of rats; J.S., Y.W., S.L., Y.B.L, D.C., and J.Z. performed the analysis of neuronal morphology and its quantitative analysis; J.S., Y.W., and S.L. collected and conducted the RNA‐seq assay, and Y.L., P.Y., and F. Y. performed the data analysis; F.Y. and Y.J.W. designed AAV‐*Ctnnd2*; J.S., Y.W., S.L,Y.J.W., S.W., and F.Y. wrote the paper; Y.J.W., S.W., and F.Y. conceived and supervised the project.

## Supporting information



Supporting Information

Supplemental Table 1

Supplemental Table 2

Supplemental Table 3

Supplemental Table 4

Supplemental Table 5

Supplemental Table 6

Supplemental Table 7

Supplemental Table 8

## Data Availability

The data that support the findings of this study are available from the corresponding author upon reasonable request.
