# Supplementary material for: Behavioral Abnormalities, Cognitive Impairments, Synaptic Deficits, and Gene Replacement Therapy in a CRISPR Engineered Rat Model of 5p15.2 Deletion Associated With Cri du Chat Syndrome
Source: Adv Sci (Weinh). 2025 Feb 18;12(14):2415224. doi: 10.1002/advs.202415224 (PMC11984882; doi:10.1002/advs.202415224)
Supplement: Supplementary file 1 — Supporting Information [file ADVS-12-2415224-s004.pdf]

ADVANCED SCIENCE
Open Access

Supporting Information

for *Adv. Sci.*, DOI 10.1002/advs.202415224

Behavioral Abnormalities, Cognitive Impairments, Synaptic Deficits, and Gene Replacement Therapy in a CRISPR Engineered Rat Model of 5p15.2 Deletion Associated With Cri du Chat Syndrome

Jingjing Shen, Yan Wang, Yang Liu, Junying Lan, Shuang Long, Yingbo Li, Di Chen, Peng Yu, Jing Zhao, Yongjun Wang, Shali Wang* and Feng Yang**

Supplementary Files

Supplementary Figures

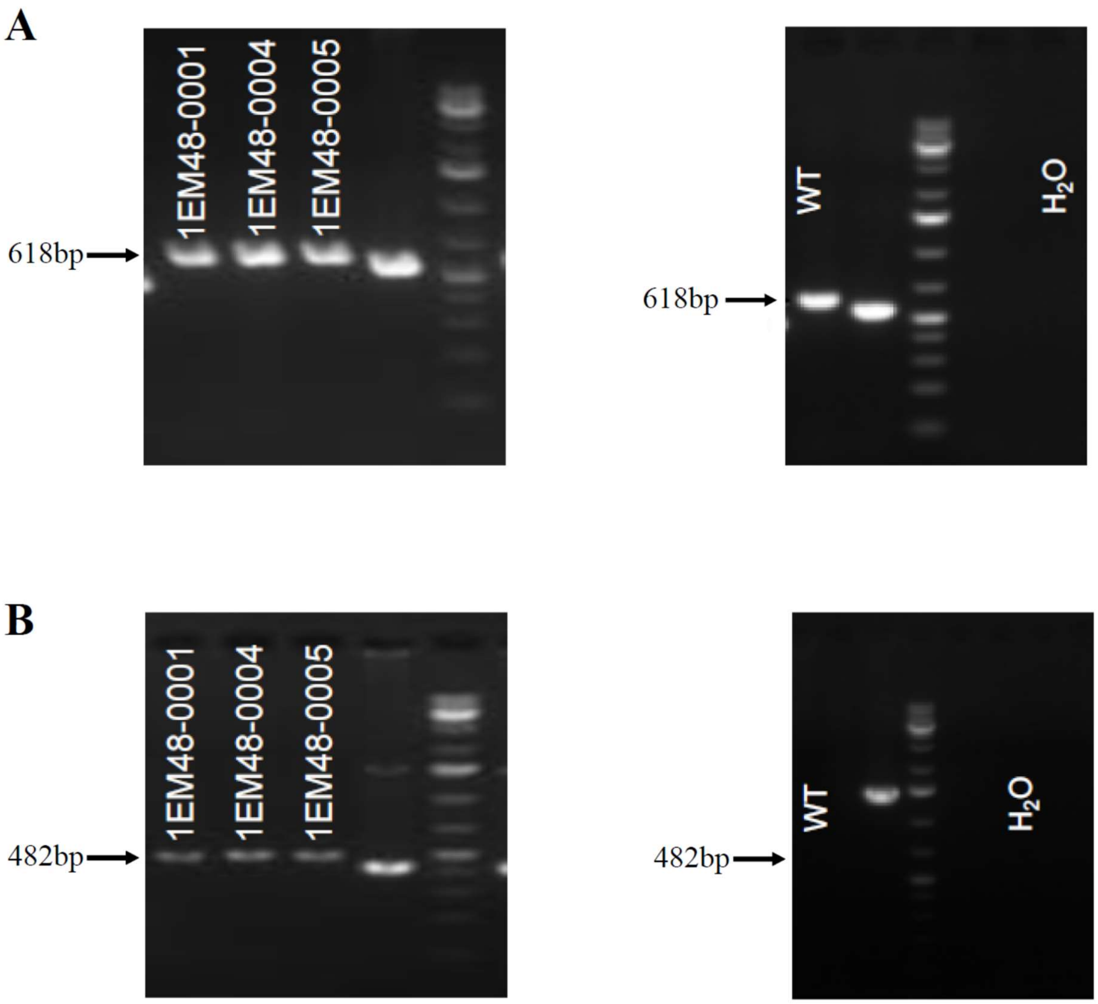

Figure S1. Confirmation of heterozygous 2q22 deletion in F1 pups via PCR product sequencing.

(**S1A** and **S1B**) These figures presented the successful confirmation of positive genotyping in three F1 generation pups (#1EM48-0001, #1EM48-0004, and #1EM48-0005) harboring a heterozygous deletion of the 2q22 region, spanning approximately 1.68 Mb of chromosomal material. The verification was achieved through PCR product sequencing, indicating the successful generation of the targeted 2q22 deletion in the Sprague Dawley (SD) rat lineage. This result highlights the effectiveness of the CRISPR-Cas9 genetic manipulation technique utilized in creation of this chromosomal deletion model associated with Cri du Chat syndrome.

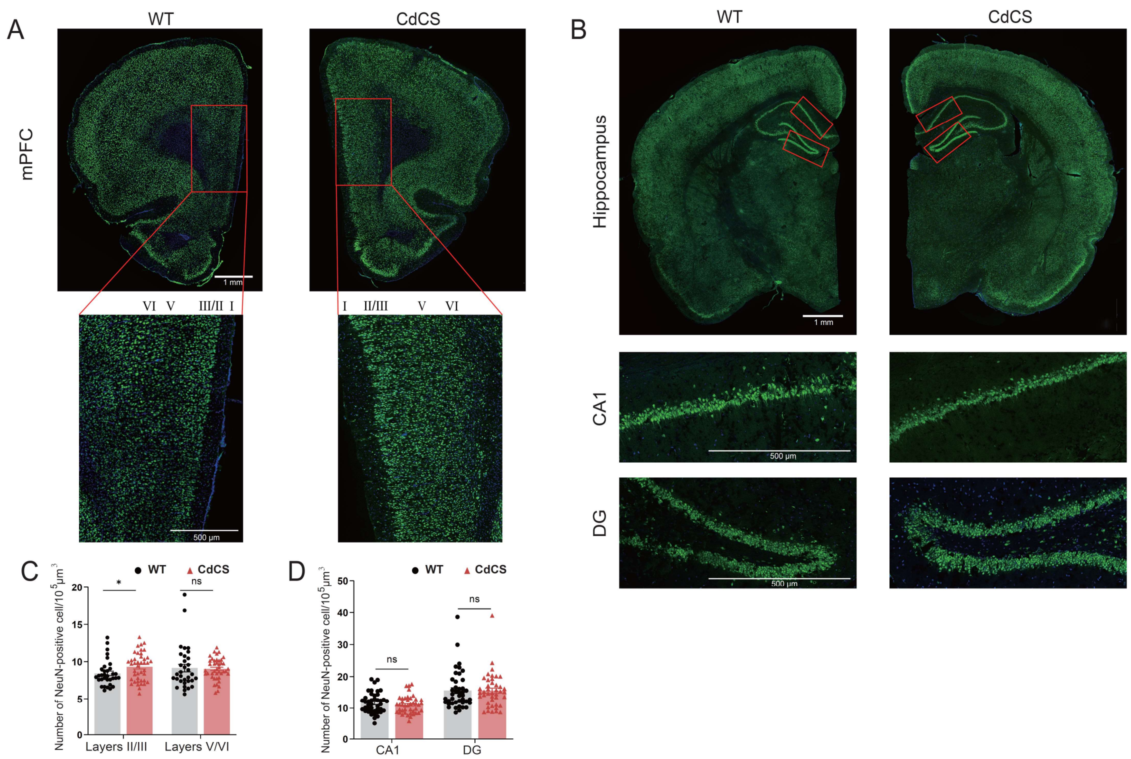

Figure S2. Pathological increase in neuronal density in the mPFC of CdCS rats.
(**A**) Representative anti-NeuN immunostained sections from the mPFC of the experimental groups. Low-magnification images, scale bar, 1 mm, high-magnification images, scale bar, 500 μm. (**B**) Representative anti-NeuN immunostained sections from the hippocampal CA1, and DG regions of the experimental groups. Low-magnification images, scale bar, 1 mm, high-magnification images, scale bar, 500 μm. (**C**) A significant increase was observed in NeuN-positive cells per 10^5 μm^3 in layer II/III of CdCS rats (9.35 ± 0.28) compared to WT rats (8.43 ± 0.30) (Mann-Whitney test, $p = 0.0255$). However, no significant difference was detected between CdCS rats (9.04 ± 0.22) and WT rats (9.15 ± 0.50) in layer V/VI (Mann-Whitney U test, $p = 0.3026$). Sample sizes: WT, n = 33 sections from 6 rats; CdCS, n = 42 sections from 7 rats. (**D**) The number of positively stained anti-NeuN neurons per 10^5 μm^3 in the hippocampal CA1 (WT: 11.62 ± 0.50; CdCS: 11.07 ± 0.43) and DG regions (WT: 15.61 ± 0.91; CdCS: 15.57 ± 0.84) did not differ significantly between WT and CdCS rats (CA1: Mann-Whitney test, $p = 0.4410$; DG: Mann-Whitney U test, $p = 0.7302$). Sample sizes: WT, n = 42 sections from 6 rats; CdCS, n = 42 sections from 7 rats. All rats were 8 weeks old. All data are presented as mean ± SEM. Statistical significance: *$p < 0.05$.

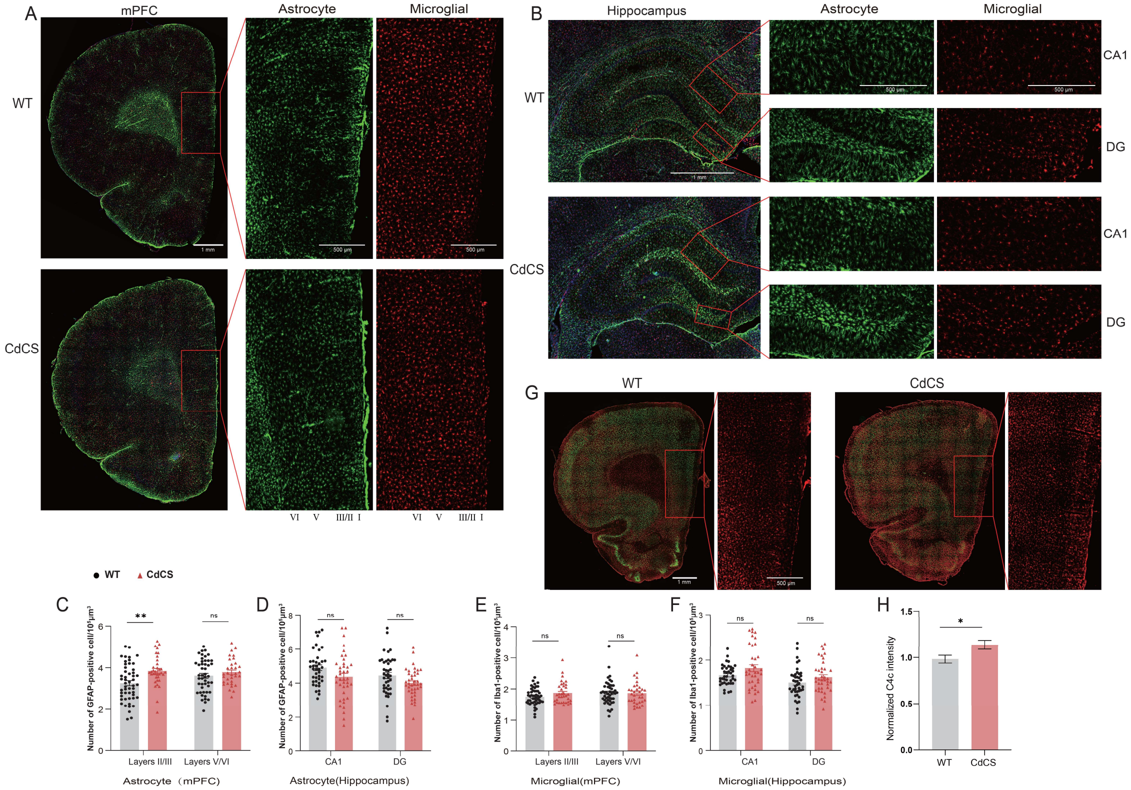

Figure S3. Increase in astrocyte density and complement C4 in the mPFC of CdCS rats. (A-B) Representative sections of the mPFC and hippocampus stained with anti-GFAP (green) and anti-Iba1(red) antibody from the experimental groups. Low-magnification images (scale bar: 1 mm) and high-magnification images (scale bar: 500 μm) were shown. (C) Quantification of positively stained GFAP (astrocyte) per 105 μm3 in the mPFC layers II/III and V/VI. There was a higher number of astrocyte cells in CdCS rats (3.84 ± 0.12) compared to WT rats (3.26 ± 0.12) in layer II/III (Mann-Whitney test, p = 0.0013), but no significant difference was observed between WT rats (3.61 ± 0.12) and CdCS rats (3.90 ± 0.10) in layer V/VI (Mann-Whitney test, p = 0.0807), indicating that there is an inflammatory response specific to the mPFC layer II/III in CdCS rats. (D) There was no significant difference in the number of GFAP-positive cells between WT (4.90 ± 0.16) and CdCS (4.36 ± 0.21) rats in CA1 (Mann-Whitney test, p = 0.064), nor between WT (4.44 ± 0.18) and CdCS rats (4.04 ± 0.13) in DG (Mann-Whitney test, p = 0.0806). (E) There was no difference in the number of Iba1-positive (microglial) cells between CdCS and WT rats in layers II/III and V/VI of the mPFC. Layers II/III: WT (1.75 ± 0.04) and CdCS (1.871 ± 0.06) rats (Mann-Whitney test, p = 0.2391), Layers V/VI: CdCS (1.868 ± 0.06) and WT rats (1.878 ± 0.06) (Mann-Whitney test, p = 0.7911). (F) There was no significant difference in the number of Iba1-positive neurons between WT (1.664 ± 0.03) and CdCS (1.821 ± 0.08) rats in CA1 (Mann-Whitney test, p = 0.2826), nor between WT (1.499 ± 0.05) and CdCS (1.620 ± 0.06) rats in DG (Mann-Whitney test, p = 0.1327). (G) Representative sections of the mPFC stained with anti-NeuN (green) and anti-C4c (red) antibody from the experimental groups. Noted: C4b is a

biologically active fragment generated from the proteolytic cleavage of C4 during its activation process. Similarly, C4c is derived from the complement C4 and represents an additional fragment or manifestation that emerges during C4's activation. Considering the present unavailability of C4b for use in animals, we employed C4c as a reliable indicator to assess the activation status of C4. Low magnification images (scale bar: 1 mm) and high-magnification images (scale bar: 500 μm) are shown. (H) There was a higher expression of complement C4c in CdCS rats compared to WT rats in the mPFC (Mann-Whitney test, $p = 0.0387$), indicating that complement C4 is in the activated condition. Sample sizes: WT, n = 21 sections from 5 rats; CdCS, n = 20 sections from 5 rats. All rats were 8 weeks old. All data are presented as mean ± SEM.

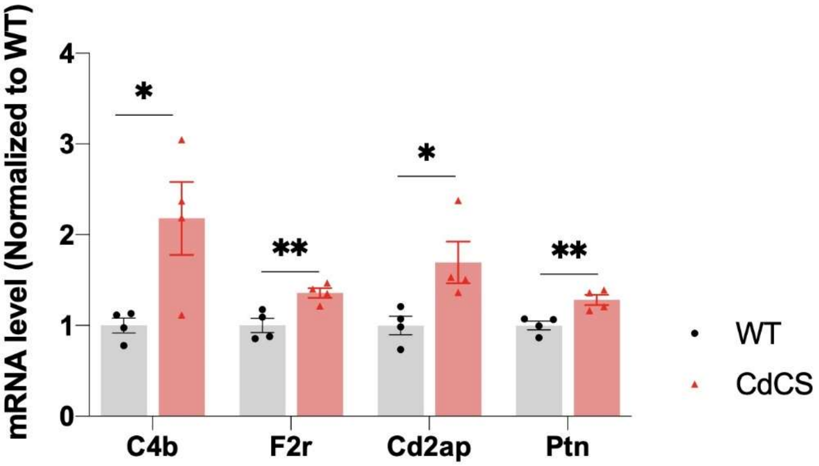

Figure S4. Validation of differential mRNA expression levels of genes associated with inflammation and innate immune responses conducted in PFC samples using qRT-PCR. The results revealed that CdCS rats exhibited significantly increased gene expression in the PFC for *C4b*, *F2r*, *Cd2ap*, and *Ptn* (*$p < 0.05$, **$p < 0.01$). (N = 4/group; 3 technical replicates per sample). Results represent mean ± SEM.

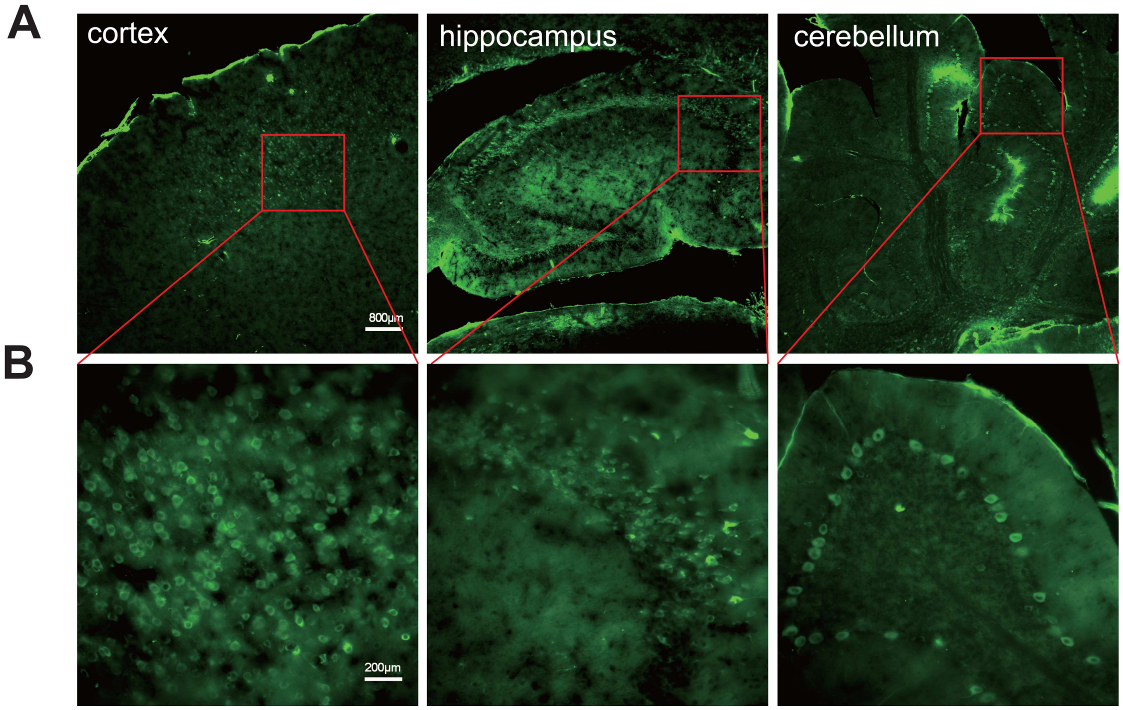

Figure S5. AAV-PHP.eB was expressed widely in multiple brain regions after 4 weeks intravenous administration.

(**A**) Representative photomicrographs of EGFP immunofluorescence under 50 x lens from the cortex, hippocampus, and cerebellum. Scale bar: 800 μm. (**B**) High magnification images of the corresponding brain region. Scale bar: 200 μm. Fluorescence images were taken after 4 weeks virus injection.

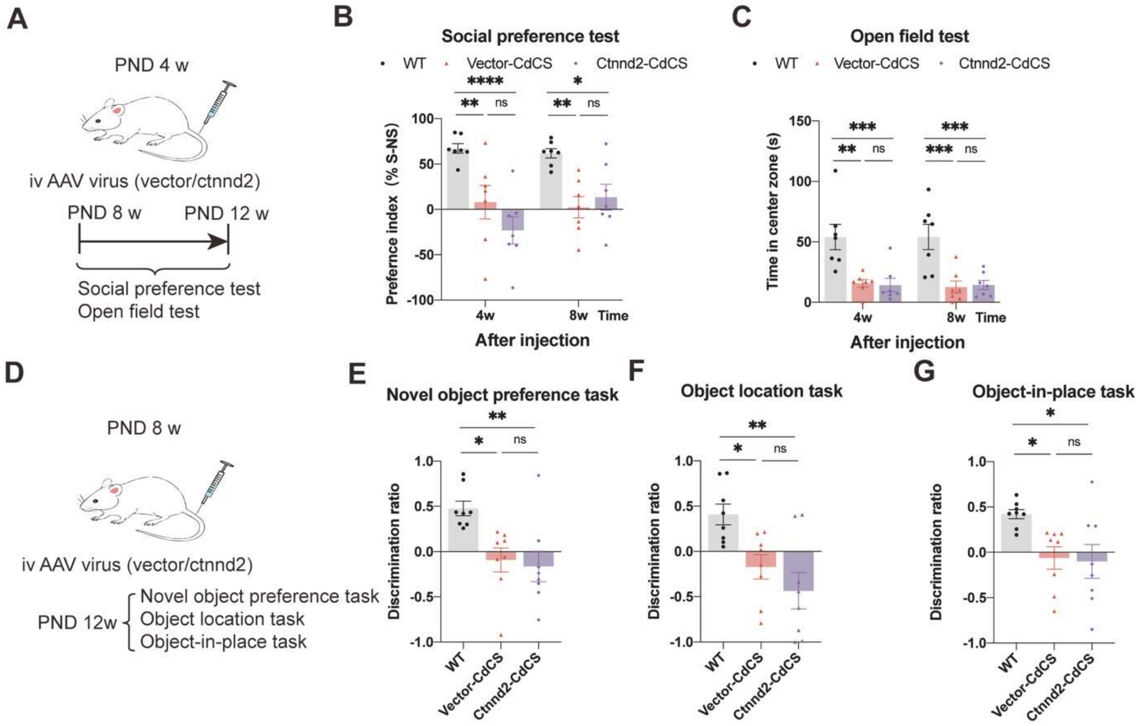

Figure S6. Effect of AAV-Ctnnd2 administration on social, anxiety-like, and cognitive behaviors in CdCS rats.

(**A**) Experimental timeline and procedures. This panel depicts the timeline of experiments conducted on WT rats, vector-injected CdCS rats (Vector-CdCS), and Ctnnd2-overexpressing CdCS rats (Ctnnd2-CdCS), following caudal intravenous injection of AAV-*Ctnnd2* at postnatal day 4 weeks (PND 4w). (**B**) Social preference test**.** Early-stage administration of AAV-Ctnnd2 (PND 4w) did not ameliorate social deficits in CdCS rats. Both Vector-CdCS and Ctnnd2-CdCS groups showed no significant improvement in social preference compared to WT rats (two-way ANOVA ($F (2, 18) = 19.75$, $p < 0.0001$). Specifically, at 4 weeks post-injection, the social preference indexes were: WT group 67.20 ± 5.29 %, Vector-CdCS group 7.99 ± 18.50 %, Ctnnd2-CdCS group -23.15 ± 15.01 %; and at 8 weeks post-injection: WT group 61.86 ± 5.05 %, Vector-CdCS group 2.31 ± 11.65 %, Ctnnd2-CdCS group 13.44 ± 14.23 %. No significant differences were observed between Vector-CdCS and Ctnnd2-CdCS groups. (**C**) Open field test. Similarly, anxiety-like behavior remained unaltered in CdCS rats after early-stage AAV-*Ctnnd2* injection. Both Vector-CdCS and Ctnnd2-CdCS groups exhibited no significant change in open field activity compared to WT rats (two-way ANOVA ($F (2, 18) = 18.31$, $p < 0.0001$). Specifically, at 4 weeks post-injection, the spent times in center zone were: WT group 53.94 ± 10.44 s, Vector-CdCS group 15.64 ± 3.08 s, Ctnnd2-CdCS group 14.21 ± 5.56 s; and at 8 weeks post-injection: WT group 54.06 ± 10.40 s, Vector-CdCS group 12.56 ± 4.95 s, Ctnnd2-CdCS group 14.31 ± 3.77 s. No significant differences were detected between the CdCS groups. (**D**) Experimental timeline and procedures (Late-Stage Injection). This panel outlines the experimental timeline for rats receiving caudal intravenous injection of AAV-*Ctnnd2* at PND 8 weeks, followed by behavioral testing at

specified ages. (**E**) Novel object preference task. Late-stage administration of AAV-*Ctnnd2* (PND 8w) failed to improve cognitive function in CdCS rats, as evidenced by the novel object preference test. One-way ANOVA (F = 7.067, p = 0.0045). Specifically, the discrimination ratio was: WT group 0.48 ± 0.08, Vector-CdCS group -0.09 ± 0.13, Ctnnd2-CdCS group -0.16 ± 0.17. N = 8 rats/group (4F+4M). (**F**) Object location task. Consistent with the novel object preference results, the object location task also indicated no improvement in cognitive function following late-stage AAV-*Ctnnd2* injection in CdCS rats. (one-way ANOVA, F = 7.793, p = 0.0029). The discrimination ratio: WT group 0.41 ± 0.12, Vector-CdCS group -0.17 ± 0.13, Ctnnd2-CdCS group -0.43 ± 0.20. N = 8 rats / group (4F+4M). (**G**) Object-in-Place task performance. The discrimination ratio: WT group was 0.42 ± 0.05, Vector-CdCS group was -0.06 ± 0.12, and Ctnnd2-CdCS group was -0.10 ± 0.19. Statistical analysis revealed significant differences among the three groups (one-way ANOVA, F = 4.867, p = 0.0183). N = 8 rats / group (4F+4M). However, no significant difference was detected between the Ctnnd2-CdCS and Vector-CdCS groups (p > 0.05), indicating that the administration of AAV-*Ctnnd2* did not effectively improve the object-in-place task performance in CdCS rats.

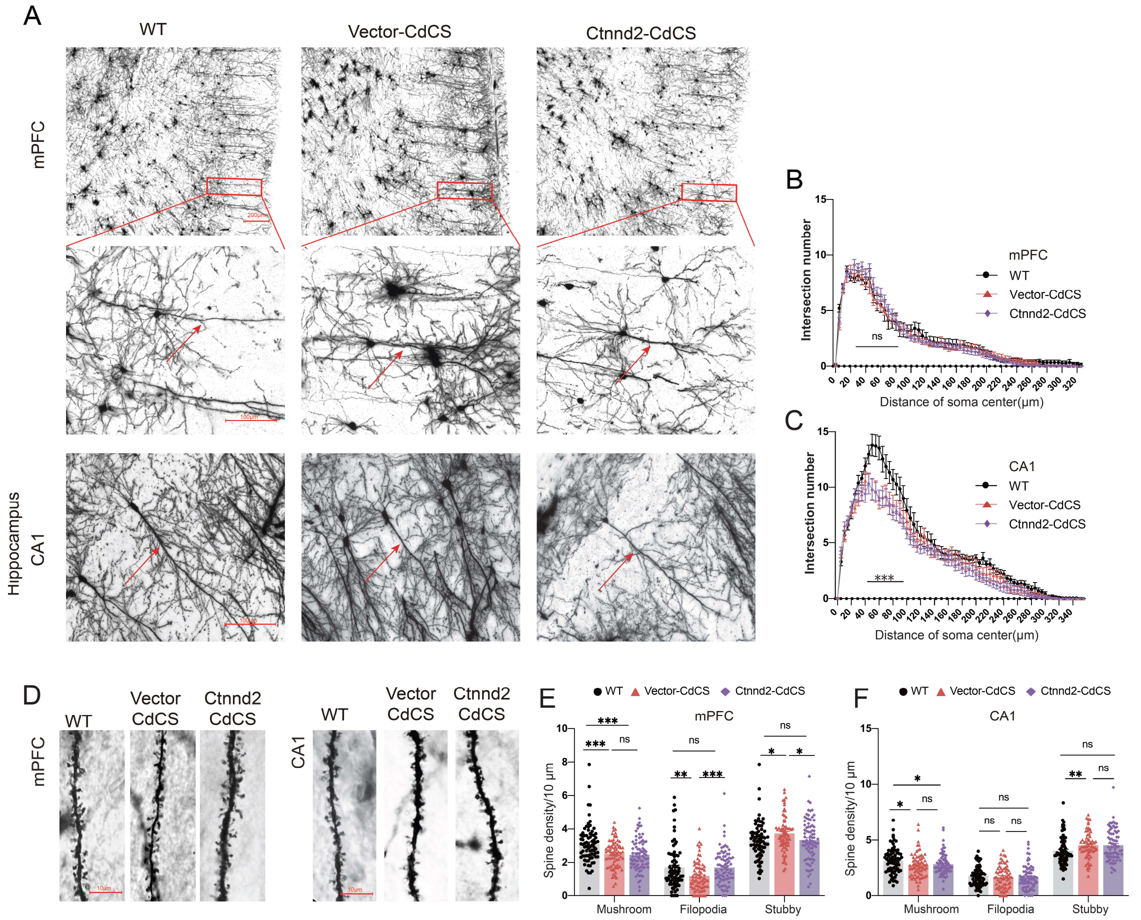

Figure S7. Partial restoration of dendritic complexity and spine density in CdCS rats following a single injection of AAV-*Ctnnd2*.

(**A**) Representative photomicrographs of Golgi-Cox-stained neurons from the mPFC and hippocampal CA1 regions of WT, Vector-CdCS, and Ctnnd2-CdCS rats. The analyzed cells are highlighted with red boxes. Low-magnification images (scale bar: 200 μm) and high-magnification images (scale bar: 100 μm) are shown. (**B**) In the mPFC region, Sholl analysis revealed no significant differences in neuronal complexity among WT, Vector-CdCS, and Ctnnd2-CdCS rats (Two-way ANOVA, $F(2, 3630) = 1.274$, $p > 0.05$, across distances of 20 μm to 250 μm). (**C**) In the hippocampal CA1 region, Vector-CdCS rats exhibited significantly fewer dendrites at distances of 45-90 μm from the soma compared to WT rats (Two-way ANOVA, $F(2, 3672) = 79.86$, $p = 0.0014$ at 45 μm, $p < 0.001$ for 50-80 μm, $p = 0.0062$ at 85 μm, $p = 0.005$ at 90 μm). However, no significant difference was observed between Vector-CdCS and Ctnnd2-CdCS rats across all distances (Two-way ANOVA, $F(2, 3672) = 79.86$, $p > 0.1$ for 20-250 μm). Sample sizes: mPFC, WT (n = 15 cells from 5 rats), Vector-CdCS (n = 19 cells from 7 rats), Ctnnd2-CdCS (n = 24 cells from 8 rats); Hippocampal CA1, WT (n = 21 cells from 7 rats), Vector-CdCS (n = 18 cells from 6 rats), Ctnnd2-CdCS (n = 18 cells from 6 rats). (**D**) Representative images of secondary dendrites from neurons located in the mPFC and hippocampal CA1 regions of WT, Vector-CdCS, and Ctnnd2-CdCS rats. Scale

bar: 10 μm. (**E**) Analysis of spine density on secondary apical branches of mPFC neurons revealed a significant reduction in mushroom-like spine density in both Vector-CdCS rats (2.51 ± 0.10, Mann-Whitney test, $p = 0.001$) and Ctnnd2-CdCS rats (2.47 ± 0.11, Mann-Whitney test, $p = 0.0002$) compared to WT rats (3.09 ± 0.13). Notably, no significant difference was found between Vector-CdCS and Ctnnd2-CdCS rats (Mann-Whitney test, $p = 0.5031$). For filopodia, a significant reduction in density was observed in Vector-CdCS rats (1.16 ± 0.10, Mann-Whitney test, $p = 0.008$) compared to WT rats (1.69 ± 0.15), while Ctnnd2-CdCS rats showed a higher density (1.70 ± 0.12) that was comparable to Vector-CdCS rats (Mann Whitney test, $p = 0.0003$), indicating a partial restoration of spine morphology. For stubby, there was a higher density in Vector-CdCS rats (3.73 ± 0.12) (Mann-Whitney test, $p = 0.0121$) compared to WT rats (3.31 ± 0.13), but a lower density in Ctnnd2-CdCS rats (3.33 ± 0.14) (Mann-Whitney test, $p = 0.0423$) compared to Vector-CdCS rats. Sample sizes: WT (n=77 branches from 5 rats), Vector-CdCS (n=75 branches from 5 rats), Ctnnd2-CdCS rats (n=84, branches from 6 rats). (**F**) In CA1 hippocampal neurons, the density of mushroom-like spines was significantly reduced in both Vector-CdCS rats (2.76 ± 0.13, Mann-Whitney test, $p = 0.03$) and Ctnnd2-CdCS rats (2.83 ± 0.11, Mann-Whitney test, $p = 0.0498$) compared to WT rats (3.16 ± 0.13). However, no significant difference was observed between Vector-CdCS and Ctnnd2-CdCS rats (Mann-Whitney test, $p = 0.6230$). For filopodia, no significant differences were found between WT rats (1.70 ± 0.09) and Vector-CdCS rats (1.67 ± 0.13, Mann-Whitney test, $p = 0.8073$), nor between Vector-CdCS rats and Ctnnd2-CdCS rats (1.64 ± 0.13, Mann-Whitney test, $p = 0.7250$). Interestingly, the density of stubby spines was significantly higher in Vector-CdCS rats (4.55 ± 0.15, Mann-Whitney test, $p = 0.0112$) compared to WT rats (4.17 ± 0.15), while Ctnnd2-CdCS rats (4.55 ± 0.16) did not differ significantly from Vector-CdCS rats (Mann-Whitney test, $p = 0.6554$). Sample sizes: WT (n = 77 branches from 5 rats), Vector-CdCS (n = 68 branches from 5 rats), Ctnnd2-CdCS (n = 73 branches from 5 rats). All rats were 8 weeks old. All data are presented as mean ± SEM. Statistical significance is indicated as follows: *$p < 0.05$, **$p < 0.01$, ***$p < 0.001$.

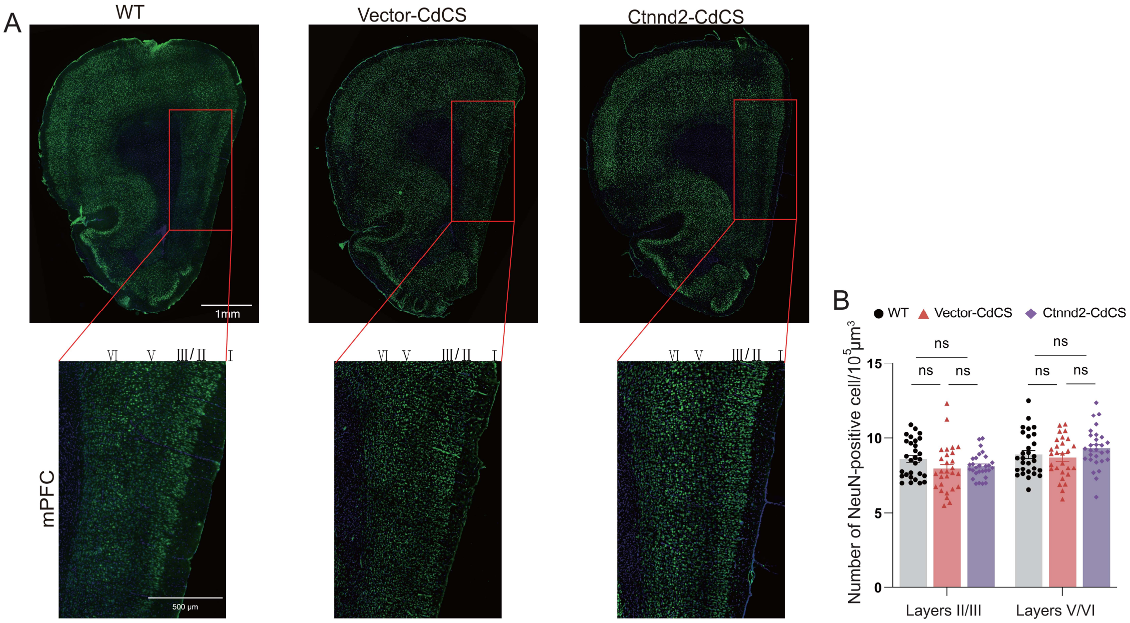

Figure S8. Neuronal density in the mPFC remains unaltered across experimental groups 4 weeks following AAV-*Ctnnd2* administration.

(**A**) Representative sections of the mPFC stained with anti-NeuN antibody from the experimental groups. Low-magnification images (scale bar: 1 mm) and high-magnification images (scale bar: 500 μm) are shown. (B) Quantification of positively stained anti-NeuN neurons per 10^5 μm^3 in the layers II/III and V/VI of mPFC. There was no significant difference in the number of NeuN-positive neurons between WT (8.62 ± 0.23) and Vector-CdCS (7.94 ± 0.30) rats in layer II/III (Mann-Whitney test, p = 0.0598), nor between Vector-CdCS and Ctnnd2-CdCS rats (8.13 ± 0.15) (Mann-Whitney test, p = 0.2643). Similarly, in layer V/VI, no significant difference was observed between WT (8.91 ± 0.26) and Vector-CdCS rats (8.71 ± 0.26) (Mann-Whitney test, p = 0.8586), nor between Vector-CdCS and Ctnnd2-CdCS rats (9.32 ± 0.24) (Mann-Whitney test, p = 0.1192). Sample sizes: WT, n = 29 sections from 5 rats; Vector-CdCS, n = 28 sections from 5 rats; Ctnnd2-CdCS, n = 29 sections from 5 rats. All rats were 14 weeks old. All data are presented as mean ± SEM.

Supplementary Table 9

Blood serum samples were analyzed to evaluate the toxicity of the AAV-PHP.eB capsid on liver and kidney functions. We compared the effects on liver and kidney functionality, as measured in 8-month-old rats, between rats that had not received AAV injection at 4 weeks of age and those that had undergone AAV injection at that age.

	Without AAV injection (N=6)	With AAV injection (N=20)	*p* value
Sex (male/female)	5/1	11/9	0.2040
Age (months)	8.67±1.76	7.45±0.64	0.4235
Glutamic pyruvic transaminase, ALT (U/L)	65.23±3.47	59.79±3.44	0.4190
Glutamic oxaloacetic transaminase, AST (U/L)	90.26±2.73	90.86±4.68	0.9463
AST/ALT ratio	1.40±0.08	1.54±0.04	0.1472
Total protein (g/L)	56.26±2.36	56.29±2.04	0.9953
Albumin (g/L)	26.32±1.06	26.83±0.93	0.7818
Globulin (g/L)	29.95±1.71	29.47±1.28	0.8515
Albumin/Globulin ratio	0.89±0.05	0.93±0.03	0.5387
Total bilirubin (μmol/L)	1.67±0.35	2.16±0.05	0.0247
Alkaline phosphatase, ALP (U/L)	194.09±29.29	173.58±18.05	0.5820
γ-Glutamyl transpeptidase, GGT (U/L)	0.93±0.13	0.67±0.12	0.2547
Creatinine (μmol/L)	25.63±1.62	25.37±0.99	0.8988
Uric acid (μmol/L)	125.82±8.68	106.79±6.25	0.1389
Urea nitrogen (μmol/L)	5.79±0.26	5.15±0.23	0.1589

The data are presented as mean ± SEM. For the comparison of sex (male/female), Fisher's exact test was employed, yielding a p-value of 0.2040. For all other comparisons, a two-tailed unpaired t-test was conducted.

Supplementary Table 10

Supplementary Table 10 | information of primer sequences

Gene	Forward primer (5’-3’)	Reverse primer (5’-3’)
Cct5	TGGAAGGCAAAGTAGGTGGG	TTCGGCATCTGTGGGTAGC
Ctnnd2	AACAGCAACAACACAGGGA	GCAGCCTTGACCACCTT
Marchf6	TGGACTGTTACTGCTGGATACC	GCTGATTGTTGTTGACCTGC
Ankrd33b	GGTTCCACTCCTGCTCCTGTTA	GCCTTCCTCTTCTCCTCCTTG
GAPDH	CAAGAAGGTGGTGAAGCAG	CAAAGGTGGAAGAATGGG
Atpsckmt	ATTCCACTTCCGGTCTCC	GGCGTTTCTCCTCTTTCC
Cmbl	CGAAGCTAACCCTTGCC	CCTGGACAACAATCACAGC
Dap	GCCCCTCAGAAAACCAG	TCCCATCTCCAGTGTGTG
C4b	ACGACCCTGACCTCCATC	ATTCCAGCTTGCCCTCAC
F2r	GCGCTTTGACGCTTTCT	TGGGCTTCTTCCCTATCAC
Cd2ap	ATTCAGCCACACCCACA	CGGTCAGTTCCAGTTCGT
Ptn	TGGAAGAAGCAGTTTGGAG	CTTGGAGATGGTGACAGTTTT

*All of primers were synthesized by Sangon Biotech (China).

Supplementary Material and Methods

Rat genotyping.
CdCS rats were genotyped routinely by isolating genomic tail DNA and performing PCR with the following primers: WT: primer sequence 5'-TTGCTCAGCTGTTAAGGGAAACTAT-3', reverse 5'-GGAGTAAGTCAACTGACTAGGGGACA-3'; CdCS: primer sequence 5'-TTGCTCAGCT GTTAAGGGAAACTAT-3', reverse 5'-AGAAGCCAAGAAGGCTCTGCCG-3'. The 618 bp wild-type and 485 bp CdCS bands were resolved on 2% agarose gels.

mRNA analysis by qPCR
Total RNA was isolated from rat PFC or HPC using Trizol reagent (Invitrogen). cDNA was generated from the tissue mRNA by reverse transcription (HIScript III RT SuperMix for qPCR, Vazyme, R323-01, China), followed by the removement genomic DNA. Quantitative RT-PCR was carried out using the CFX Real-Time PCR Detection System (Bio-Rad). Each reaction was run in triplicate and analyzed following the ΔΔCt method using GAPDH as a normalization control. Primers for all target genes are listed in Supplementary Table 10.

Immunoblotting
The protein was extracted using a RIPA lysis buffer (P0013B, Beyotime) and PMSF (ST506, Beyotime) in glass homogenizer. The homogenate was incubated on ice for 30 min and followed by centrifugation at 12,000, 4°C for 15 min. After centrifugation, the supernatant was collected, boiled in 5 × SDS loading buffer (P0015, Beyotime) for 5 min, separated on 10% SDS-PAGE, and transferred to PVDF membranes (Millipore, IPVH00010). Membranes were blocked with 5% non-fat dry milk dissolved in TBST for 2 hours at room temperature with gentle shaking, and then incubated with primary antibodies: anti-δ-catenin (Abcam, ab184917, 1:1000), anti-GAPDH (Proteintech 10494-1-AP, 1:4000), at 4°C overnight followed by corresponding horseradish-peroxidase (HRP)-conjugated secondary antibodies. Protein expression was detected by ChemiScope 6200 Touch (CLINX, China) and intensities of bands were quantitated by ImageJ software.

Physical development assays
Observers blinded to grouping. The test time was as fast as possible to shorten the separation time from the mother rats. During the test, the pups were always kept on a constant temperature mat to avoid losing temperature. Newborn rats were assessed development milestones including body weight, body length and tail length, auricle detaching and eyelid opening [1]. Auricle detaching: the ears of pups separated completely from their heads. Eyelid opening: the eyes of pups opened more than or equal to half of the normal eye fissure. *Neuromuscular reflex development***.** It was assessed for surface righting, air righting and negative geotaxis [2]. Surface righting: pups placed supine to return to prone position with all 4 paws on the ground within 4 seconds. Air righting: pups released upside down from a height of 30 cm and land on all 4 paws on a bed of shaving. Negative geotaxis: pups were

placed head down on a 30° wooden slope and turn 180° into a head-up position within 30 seconds.
Sense function. It was assessed for auditory response and olfactory response[3]. Auditory response: pups respond with a quick body curl or body tremor after a small metal object is dropped on a lab bench 15 cm from the pups. Olfactory response (the homing test): pups were transferred to a test cage layered one-third with clean and one-third with soiled bedding from the home-age. The pup was placed in the middle of the chamber and allowed to wander. Homing was considered successful if the pup moved over and stayed in the portion of the cage that contained soiled home-cage bedding.
Rope grip test. It was used to assessed muscle strength [4]. Animals were placed midway on a string between two supports with a 2 cm thick padding under it to protect animals and rated as follows: 0, falls off, the suspension time is less than 10 seconds and fall off, it will be calculated as 0; 1, Hangs onto string by one or both forepaws; 2, Same as for 1, but attempts to climb onto string; 3, Hangs onto string by one or both forepaws plus one or both hindpaws; 4, Hangs onto string by forepaws and hind paws plus tail wrapped around string; and 5, Escapes to the supports. The suspension time is timed for 60 seconds, and if animals escape to the supports, it is also recorded as suspension for 60 seconds. The final score was the average of 3 trials. The interval between each trial was 20 min.
Beam walking test. It was used to assess the motor coordination and balance ability[5]. A beam (2 cm wide) was placed 50 cm above the floor with one end in a box (non-transparent, $15 \times 15 \times 15\ cm^3$). There is a 2 cm thick padding under it to protect animals. Padding material from cages was placed in the box to attract the animal to walk through a 60 cm long distance on the beam. Rats were placed at the end without box and trained three times per day for 3 days before testing. The percentage of crossing beam, the time to cross beam and the numbers of hindlimb slipping were recorded and measured. the result was the average of 3 trials. The interval between each trial was 20 min.
Gait analysis. It was used to study locomotor behavior[6]. The animals' paws were dipped in ink (forepaws: red ink; hind paws: blue ink), as the animal walked across white paper, the footprints were left on it, then the stride length and stride width were analyzed manually. For each animal, three sets of footprints were generated from three separate traverse of the track.

Behavioral assays

All behavioral experiments were conducted between 2 p.m. and 10 p.m. All measures of exploration were made with the experimenter blind to the group of each animal. The arena was cleaned thoroughly with a 75% ethanol solution between each rat to minimize odor cues. all tests were conducted in a dimly lit room except for the elevated plus maze. Animals were be taken to the behavioral room three days in advance to habituate environment, with an interval of three or four days between different behavioral test. A video camera was suspended above the arena, then the videos were evaluated by using Smart v3.0.06 video tracking system (Panlab, Harvard apparatus).
Self-grooming test. Self-grooming test was used to assess repeat stereotypical actions. Rats were placed in a gray 100×100×48 cm opaque box and moved freely for 10 min. Cumulative time spent grooming were scored for each rat.

Open field test. The open field test was used to assess locomotor activity and anxiety-like behavior[7]. the arena was 100 × 100 × 48 cm (Length × Width × Height) with opaque walls. One rat at a time was placed in the center of the arena, and allowed to freely move for 10 min. The distance moved, time spent in the center zone and entries into the center zone were measured.

Elevated plus maze. The elevated plus maze was conducted to assess anxiety-like behavior[7]. The apparatus consists of two open arms without walls and two closed arms with 50 cm high walls, with a square area (diameter: 10 cm) leading to the four arms in the center. The entire maze was elevated 50 cm from the floor. Rats were placed in the central area of the square, facing toward the open arm, and allowed to freely move for 5 minutes, the distance moved, the time spent in open arm, and the open arm entries were counted.

Social preference test. A three-chamber social-interaction assay was performed to assess social deficits [8]. The apparatus contained three chambers (40 × 40 × 40 cm, Length × Width × Height) with doorways allowing for access to each chamber. There was a cylindrical mesh wire cage (Diameter: 20 cm, Height: 30 cm) in the left and right chamber respectively. The test was conducted in two phases with different stimuli in the cages. The first phase is two identical bottles (nonsocial stimulus) placed in the cages, the test animal was placed in the middle chamber and admitted exploring freely for 10 min habituation period. then the subject was returned to its home cage and had a 5 min rest. Following this, A new bottle (nonsocial stimulus, NS) was placed in one of the side cages together with an age-sex matched rat (social stimulus, S) in the other side cages. The rat was allowed to explore for another 10 min in the second phase. the amount of time that the rat spent sniffing each wire cage was quantified and the preference index was calculated as $(T_S-T_{NS})/(T_S+T_{NS}) \times 100\%$. T_S and T_{NS} represent the time spent exploring the nonsocial stimulus and social stimulus, respectively.

Behavioral test of cognitive function. It includes three detection, novel object preference task, object location task and object-in-place task [9]. The animals were habituated to the arena without stimuli for 10~15 min daily for 3 days before the testing， the experimenter handles the animals daily 2 min over the course of 3 days prior to testing to reduce stress stimulation. The arena was 100 × 100 × 48 cm (Length × Width × Height) with opaque walls. The stimuli objects were a series of bottles and blocks with varied in shape, color, and size, bottles: 25 × 8 cm (Height × Diameter); cone: 6 × 8 cm (Height × Diameter); cube: 8 × 8 × 8 cm (Length × Width × Height). All of them were too heavy for the animals to displace and were placed near the corners on one wall in the arena (15 cm from each adjacent wall). Each test consists of an acquisition phase and a recognition test with an interval of 4 hours between the two phases. Exploring behavior is defined as an animal pointing its nose directly towards the target, and the distance is less than 2 cm. Other behaviors, such as sitting or leaning against objects and looking around, are defined as non-exploratory behaviors. Discrimination between the objects was calculated using a discrimination ratio, calculated as the difference in the time spent exploring the novel and familiar objects divided by the total time spent exploring the objects.

Novel object preference task. This test was performed to assess the ability of novel object recognition. In acquisition phase, two identical objects are respectively placed 15cm away

from one side wall. Animals are placed in the open field facing the middle of the opposite wall, allowing animals to explore freely for 5 minutes. All the objects were cleaned with alcohol to remove olfactory cues. After 4 hours, one of object was replaced by a new object. Put animals in the same position as before and record for 5 minutes. Discrimination ratio = $(T_{novel} - T_{old}) / (T_{novel} + T_{old})$, T_{novel}: the time of exploration novel object; T_{old}: the time of exploration old object.

Object location task. This test was performed to assess the ability of location recognition. In acquisition phase, the rats were exposed to another two identical objects (C1 and C2), Animals are placed in the open field facing the middle of the opposite wall and permitted to explore the objects for 5 min. All the objects were cleaned with alcohol to remove olfactory cues. After 4 hours, C1 was placed in the same position. C2 was positioned it diagonally at C1. The rats were placed in the same place as before and explored freely for 5 min. Discrimination ratio = $(T_{C2} - T_{C1}) / (T_{C1} + T_{C2})$, T_{C1}: the time of exploration C1; T_{C2}: the time of exploration C2.

Object-in-place task. In acquisition phase, four different objects (A, B, C and D) were placed in the corners of the arena 15 cm from the walls. The rat was placed in the center of the arena and explored 5 min. all the objects were cleaned with alcohol to remove olfactory cues. After 4 hours, B and D were exchanged the position, the rats were placed into the arena and be free to explore for 5 min. Discrimination ratio = $(T_{B+D} - T_{A+C}) / T_{A+B+C+D})$, T_{B+C}: the total time of exploration B and D; T_{A+C}: the total time of exploration A and C. $T_{A+B+C+D}$: the total time of exploration A, B, C and D.

Preprocessing of RNA sequencing data

Raw sequencing reads were quality-checked through the utilization of the fastp software tool [10], aimed at eliminating adapter sequences and trimming low-quality bases. Clean reads were then aligned to the reference genome using Hisat2 v2.0.5 [11]. To quantify the gene-level read counts, featureCounts v1.5.0-p3 [12] was employed, and the Fragments Per Kilobase of transcript per Million mapped reads (FPKM) of each gene was calculated based on the length of the gene and reads count.

Differential expression analysis

The raw count data were analyzed using the DESeq2 v1.38.3 package in R [13]. Genes were considered for further analysis if they had a minimum expression of 10 reads in at least half of the samples across conditions. The DESeq() function was utilized to perform differential expression analysis, adjusting for batch effects. Genes were deemed differentially expressed based on significant fold changes (log_2FoldChange) and adjusted P-values (p.adj). We refined our analysis by excluding genes with low expression levels, retaining 14,648 genes in the PFC and 14,608 genes in the hippocampus of CdCS rats compared to their wild-type counterparts, and 14,659 genes in the PFC and 14,633 genes in the hippocampus of CdCS rats across pre- and post-AAV-Ctnnd2 administration conditions. We considered genes significant in the PFC if their adjusted p-value (p.adj) was less than 0.01 and in the hippocampus if p.adj was less than 0.1. Genes were classified as upregulated or downregulated based on thresholds exceeding $log_2FC > 0.22$ or $log_2FC < -0.22$, respectively.

Gene set enrichment and Venn diagram

We conducted gene set enrichment analyses by mapping the Ensembl Gene IDs of marginally significant genes (with an FDR p-value < 0.05), categorizing them based on genotype directionality, and utilizing the Gene Ontology database (https://geneontology.org/). Our analysis encompassed the assessment of enrichment within three distinct gene sets: Gene Ontology Biological Processes (GO-BP), Gene Ontology Molecular Function (GO-MF), and Gene Ontology Cellular Component (GO-CC). Furthermore, we leveraged a specialized tool available at https://bioinformatics.psb.ugent.be/webtools/Venn/ to calculate and generate customized Venn diagrams, visually illustrating the intersections and unique contributions of these gene sets.

Reference

[1] U. J. Hyoung, Y. J. Yang, S. K. Kwon, J. H. Yoo, S. C. Myoung, S. C. Kim, Y. P. Hong, *J Prev Med Public Health* **2007**, *40* (2), 155, https://doi.org/10.3961/jpmph.2007.40.2.155.

[2] J. Y. Wu, K. A. Henins, P. Gressens, I. Gozes, M. Fridkin, D. E. Brenneman, J. M. Hill, *Peptides* **1997**, *18* (8), 1131, https://doi.org/10.1016/s0196-9781(97)00146-0.

[3] A. Sarkar, K. Balogun, M. S. Guzman Lenis, S. Acosta, H. T. Mount, L. Serghides, *PLoS One* **2020**, *15* (11), e0242513, https://doi.org/10.1371/journal.pone.0242513.

[4] M. Munakata, H. Shirakawa, K. Nagayasu, J. Miyanohara, T. Miyake, T. Nakagawa, H. Katsuki, S. Kaneko, *Stroke* **2013**, *44* (7), 1981, https://doi.org/10.1161/strokeaha.113.679332.

[5] F. Wang, Y. J. Yang, N. Yang, X. J. Chen, N. X. Huang, J. Zhang, Y. Wu, Z. Liu, X. Gao, T. Li, G. Q. Pan, S. B. Liu, H. L. Li, S. P. J. Fancy, L. Xiao, J. R. Chan, F. Mei, *Neuron* **2018**, *99* (4), 689, https://doi.org/10.1016/j.neuron.2018.07.017.

[6] C. C. Chan, K. Khodarahmi, J. Liu, D. Sutherland, L. W. Oschipok, J. D. Steeves, W. Tetzlaff, *Exp Neurol* **2005**, *196* (2), 352, https://doi.org/10.1016/j.expneurol.2005.08.011.

[7] M. Yu, L. Ma, Y. Yuan, X. Ye, A. Montagne, J. He, T. V. Ho, Y. Wu, Z. Zhao, N. Sta Maria, R. Jacobs, M. Urata, H. Wang, B. V. Zlokovic, J. F. Chen, Y. Chai, *Cell* **2021**, *184* (1), 243, https://doi.org/10.1016/j.cell.2020.11.037.

[8] a) B. Rein, K. Ma, Z. Yan, *Nat Protoc* **2020**, *15* (10), 3464, https://doi.org/10.1038/s41596-020-0382-9; b) L. Qin, K. Ma, Z. J. Wang, Z. Hu, E. Matas, J. Wei, Z. Yan, *Nat Neurosci* **2018**, *21* (4), 564, https://doi.org/10.1038/s41593-018-0110-8.

[9] a) G. R. Barker, E. C. Warburton, *J Neurosci* **2011**, *31* (29), 10721, https://doi.org/10.1523/jneurosci.6413-10.2011; b) J. K. Denninger, B. M. Smith, E. D. Kirby, *J Vis Exp* **2018**, (141), https://doi.org/10.3791/58593.

[10] S. Chen, Y. Zhou, Y. Chen, J. Gu, *Bioinformatics* **2018**, *34* (17), i884, https://doi.org/10.1093/bioinformatics/bty560.

[11] D. Kim, J. M. Paggi, C. Park, C. Bennett, S. L. Salzberg, *Nat Biotechnol* **2019**, *37* (8), 907, https://doi.org/10.1038/s41587-019-0201-4.

[12] Y. Liao, G. K. Smyth, W. Shi, *Bioinformatics* **2014**, *30* (7), 923, https://doi.org/10.1093/bioinformatics/btt656.

[13] M. I. Love, W. Huber, S. Anders, *Genome Biol* **2014**, *15* (12), 550, https://doi.org/10.1186/s13059-014-0550-8.
